# Parametric Modelling of the Crystalline Microstructure of the MCM41-Type Mesoporous Silica Modified with Derivatives of Alkyls

**DOI:** 10.3390/ma17133065

**Published:** 2024-06-21

**Authors:** Jarosław Stocki, Marcin Kuśmierz, Weronika Sofińska-Chmiel, Marek Stankevič, Marcin Puchała, Marek A. Kojdecki, Robert Gąska, Henryk Grajek

**Affiliations:** 1 Faculty of Advanced Technologies and Chemistry, Military University of Technology, 00-908 Warsaw, Polandhgrajek5819@wp.pl (H.G.); 2 Analytical Laboratory, Institute of Chemical Sciences, Faculty of Chemistry, Maria Curie Skłodowska University in Lublin, 20-031 Lublin, Poland; marcin.kusmierz@mail.umcs.pl (M.K.);; 3 Department of Organic Chemistry and Crystallochemistry, Institute of Chemical Sciences, Faculty of Chemistry, Maria Curie-Skłodowska University in Lublin, 20-614 Lublin, Poland; 4 Institute of Mathematics and Cryptology, Military University of Technology, 00-908 Warsaw, Poland

**Keywords:** siliceous mesoporous adsorbents, immobilized guest structures, crystalline microstructure, inverse problems

## Abstract

A siliceous material in which a framework order was established with a surfactant with sixteen carbon atoms in alkyl chains, MCM-41-C16, was synthesised, surface-modified, and tested regarding the selected physical properties. The pristine material was extracted in an acidic aqueous alcohol and then lined with different surface groups. The properties of four adsorbents were investigated using XRD, X-ray photoelectron spectroscopy, and N2 physisorption techniques. The unit–cell constant was determined from X-ray diffractograms, being in fixed relation to the edge length of the hexagonal frame. The specific surface areas of mesopores and whole crystallites were determined from low-temperature N2-physisorption isotherms. The novelty of this work is a mathematical model of a crystalline microstructure explaining the sizes and shapes of crystalline grains in relation to adsorption features, proposed and successfully tested with the aforementioned experimental data. The roughness of the surface is different from one that is necessary to explain the experimental characteristics quantitatively.

## 1. Introduction

In our examination, attention was given to siliceous mesoporous materials, mainly due to their elevated surface areas, defined pore sizes, and ability to interact with atoms, ions, and nanoparticle molecules, both on the surface and inside their pores, which makes them promising materials for several applications, such as environmental remediation. Siliceous mesoporous structures are commonly prepared by self-assembling inorganic precursors in solutions with structure-directed quaternary ammonium ions. Over the years, different sources of precursors, structure drivers, and changes during and after synthesis have been evaluated. In the early 1990s, researchers at Mobil Oil Corporation developed a new family of mesoporous materials (i.e., MCM-41, a Mobil code for mesoporous catalytic purposes) [1]. The beneficial features of these materials are high surface areas, narrow pore size distribution, and uniform pore size, typically in the range of 3–10 nm. Traditionally, three mesophases, collectively known as MCM-41 adsorbents, were synthesized and are described as mesoporous (according to the IUPAC nomenclature) due to their pore diameters ranging between 2 and 50 nm [2]. The MCM-41 family presents three mesoporous materials, silicates, and aluminosilicates with different pore arrangements [1,3,4]: (1) MCM-41-hexagonal two-dimensional (2D) structure with 6 mm space-group symmetry; (2) MCM-48-cubic three-dimensional (3D) system with Ia3d space-group symmetry; and (3) MCM-50–lamellar structure without space-group symmetry. Perhaps the most widely recognized and used ordered mesoporous silica adsorbent is known as MCM-41 [5,6]. The most exciting features of these materials, such as a relatively large BET-specific surface area and pore volume, hydrophobic surface nature, etc., manifest themselves as selective adsorbents for removing volatile organic compounds present in high-humidity gas streams or wastewater.

Some authors argue that mesoporous siliceous adsorbents have both the amorphous characteristics of gels and the well-ordered zol structure of crystalline materials [7,8,9]. The framework of MCM-41-type adsorbents usually consists of hexagonally arranged cylindrical mesopores with a large surface area and narrow pore size distribution. Surface modification can be conducted in various ways, such as esterification and covalent attachment of the functional groups. Modification of the surface of MCM-41 adsorbents by organic groups obtained by silylation is the commonly used method for the preparation of hybrid organic–inorganic materials [6]. The reaction of the solid substrate with suitable silylating agents leads to covalent bonding between the organic and inorganic components. The immobilisation of organic groups in the readily accessible mesopores of the pristine MCM-41 provides a way to skillfully act on the chemical and physical properties of the materials without compromising their basic geometry and mechanical strength [1,10,11,12].

MCM-41 silica material has silica tetrahedra terminated in either oxy–silica bridges or siloxane groups present on the surface of pristine MCM-41 or with selected alkyl derivatives immobilised by the rest of the organic compounds and water used in the synthesis processes. In the course of the reaction, during the first stage, the surface of the formed silica is covered with a layer of water, which is removed from the silica surface because of the deposition of alkyl derivatives. Grajek et al. [13] recently showed that the covalent attachment of EDA self-assembled monolayers within a mesoporous structure of SBA-15 could create a hierarchical ligand array capable of generating charge transfer complexes.

The type and functionality of the alkylsilane employed for surface modification are crucial. Modification of the surfaces of the ordered hexagonal array of parallel silica mesotunnels with alkyl chains provides the possibility to tailor the accessible pore size of the mesoporous solids, increasing the surface hydrophobicity or passivating the silanol groups, thereby protecting the framework against hydrolysis. The diameter of the pores can be progressively decreased by employing alkyl chains of different lengths and functionalities [14]. By chemically attaching organic silylating species, the surface chemistry of pristine MCM-41 can be enhanced to increase its hydrophobic properties. The effectiveness of the modifications is attributed to the free and germinal silanol groups over MCM-41 surfaces.

The liquid crystal-controlled synthesis of MCM-41 provides various synthesis methods to improve new materials. Modified synthesis methods and the use of liquid crystal chemistry provided by a precisely selected surfactant can effectively prepare new porous materials.

A high number of potential surface binding sites usually accompanies an increase in the surface area of mesoporous materials. Apart from the covalent attachment of the organic components at the outer particle surface, the relatively large pore size gives access to binding sites in the pore interior. As a result, materials with enhanced separation kinetics, high material loading, and excellent separation selectivity can be obtained [15].

The covalent attachment of organic moieties to the siliceous surface can also be called immobilization and is carried out in two fundamental ways: co-condensation [16] and lining, as we presented in the case of SBA-15 [13]. In this context, it is necessary to add that the co-condensation method is usually regarded as a one-pot route, which involves the addition of the organic group, usually a siliceous material, with $SiO_{4}^{4-}$ tetrahedra containing reactive alkoxy groups created during a mesoporous process carried out uniquely. This route traditionally led to functionalized silica adsorbents with both the outer grain surface and the inner surface of the pores [2,3,4,5].

This co-condensation method is called one-pot. The method is similar to the synthesis of MCM-41, in which trialkoxyorganosilane species are incorporated into a TEOS (or TMOS) solution and hydrolyzed as well as condensed in the presence of morphological form-directing agents or templates. During the synthesis of MCM-41 materials, this method affects the surface density of silanol [17]. Typically, a higher concentration of silanol groups is desirable because they act as active sites to anchor the organic groups to the surface. However, it should be known that many silanols are lost at higher temperatures as a result of condensation reactions.

The silica source can also be subject to mild hydrothermal conditions in the presence of a micelle-forming cationic detergent. The adjustable alkaline pH of silica condensation can be provided with an aqueous solution of a base such as NaOH, NH_4_OH.

Some authors [18,19,20] synthesized the MCM-41 family samples employing alkyl trimethylammonium bromide surfactants with different units of (CH_2_) 2, of 10, 12, 14, 16, and 18 carbons as structure-directing agents in a self-ordering hydrothermal system to obtain various pore sizes tunable from 2.41 to 4.24 nm. The X-ray diffraction patterns from MCM-41 silicas usually contain several peaks and can be indexed by assuming an ordered hexagonal array of parallel silica mesotunnels [5,21,22,23]. For example, MCM-41 adsorbents prepared with cationic cetyltrimethylamonium (CTA^+^) surfactants produce X-ray reflections due to the ordered hexagonal array of parallel silica mesotunnels. It is also known that the MCM-41 molecular sieve, which belongs to the M-41(S) family, exhibits a hexagonal arrangement of uniform almost-cylindrical pores and is especially suitable as a siliceous model adsorbent [2,19,20,24].

The synthesis of MCM-41 can be performed in several procedures, which differ mainly in the pH of the reaction mixture and the silica source. Amphiphilic templating agents, e.g., alkyl trimethylammonium halogenides, are used with silica precursors such as tetraalkoxysilanes or colloidal silica. After the reaction, the template is removed by calcination at 823 K. MCM-41 offers unique properties as an adsorbent in separation science and technology. However, the material obtained by Beck et al.’s classical procedure [1,18,25] yields loose agglomerates with a wide particle size distribution. Agglomerates are composed of primary particles of about 50–100 nm [26]. As a consequence, grinding and sorting into specific narrow cuts produces a large number of small particles [27], and the control of the morphology of MCM-41 particles, i.e., the synthesis of balls of a specific size, provides new possibilities for using MCM-41, e.g., as a filling material in chromatography. So far, only hexagonal arrangements of the MCM-41 structure have been described in the literature [2,23,28]. The synthesis procedure is a modification of the known synthesis of monodisperse silica beads by Stöber [29,30], which involves the hydrolysis of tetraalkoxysilane in a mixture of low-boiling alcohol and an aqueous ammonia solution.

The formation of monolayers with silanes might appear to be attractive, since it provides closely packed and highly ordered monolayers with enhanced stability. However, attention must be paid to undesired vertical polymerization [31,32]. On the other hand, surface modification using monofunctional silanes gives reproducible surface coverages where vertical polymerization is avoided. The attachment of organic components can modify the physical and chemical properties of mesoporous silica materials either on the outer silica surface or in the interior of the channels. In previous years, MCM-41 materials were modified with long chains (e.g., C 18), and the obtained products showed a significant reduction in surface area, pore volume, and pore diameter [33]. The molecular length of the silylation reagent is crucial for covering the surface with organic components. Due to steric hindrance, longer chains bind mainly to the external surface. Only a few chains are attached to the mesopores, leaving a significant portion of surface silanol groups unreacted. These residual silanol groups are capable of forming weakly acidic groups.

Over the years, different sources of precursors, structure drivers, and changes during and after synthesis were evaluated [20]. These materials have high surface area values and well-defined pore sizes. Introducing organic groups in the easily accessible pores of MCM-41 provides a way to manipulate the chemical and physical properties of these materials without compromising the basic geometry and mechanical strength. The surface can be modified chemically (covalent attachment) or physically by adsorption of the functional group [34]. The framework of MCM-41 silica material has SiO_2_ tetrahedra terminating in either silica bridges (
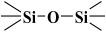
) or siloxane groups (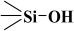) on the mesotunnel surfaces. Both groups are prone to functionalization, although reaction with silanols is considered the main modification pathway [20].

## 2. Materials and Methods

### 2.1. Essential Materials

Sodium hydroxide (NaOH), Belle chemical, Billing, MT, USA;

Cetyltrimethylammonium Bromide (C_16_H_33_N(CH_3_)_3_Br), Merck, Darmstadt, Germany;

Tetraethyl orthosilicate ((C_2_H_5_O)_4_Si), Sigma Aldrich Chemistry, Steinheim, Germany;

3-aminopropyltriethoxysilane [H_2_N-CH_2_CH_2_CH_2_-Si≡(OC_2_H_5_)_3_], Sigma Aldrich, Steinheim, Germany;

3-mercaptopropyltriethoxysilane [HS-CH_2_CH_2_CH_2_-Si≡(OC_2_H_5_)_3_], Sigma Aldrich Steinheim, Germany;

propyltriethoxysilane [HS-CH_2_CH_2_CH_2_-Si≡(OC_2_H_5_)_3_], Sigma Aldrich, Steinheim, Germany.

In the present study, we have struggled to provide a systematic report on the preparation and comparable characterization of the honeycomb (hexagonal) MCM-41 lined with organized monolayers of functional molecules covalently bound to the mesoporous support with aminoalkylsilyl, thioloalkylsilyl, and alkylsilyl groups (C3). We aimed to determine which changes would occur after attaching different functional groups to the mesoporous structure of MCM-41S. For this purpose, about 36.5 cm^3^ of redistilled water, 8 cm^3^ of a 5 M aqueous NaOH solution, and approximately 7.8 g of CTA^+^Br^−^ (i.e., C_16_H_33_(CH_3_)_3_N^+^Br^−^) surfactant as a micelle builder were introduced into an Erlenmeyer flask. This solution was stirred for about 0.5 h. At the same time, a mixture of the appropriate modifier and tetraethyl orthosilicate (TEOS) in stoichiometric relationship was prepared in the following amounts:

1. Basic → TEOS = 14 cm^3^;
2. n-Propyl-SAMMS TEOS: *n*-propylotriethoxysilane [CH_3_CH_2_CH_2_-Si≡(OC_2_H_5_)_3_] = 14 cm^3^: 1.7 cm^3^;
3. Amina-SAMMS TEOS: 3-aminopropyltriethoxysilane [H_2_N-CH_2_CH_2_CH_2_-Si≡(OC_2_H_5_)_3_] = 14 cm^3^: 1.7 cm^3^;
4. Thiol SAMMS TEOS: 3-mercaptopropyltriethoxysilane [HS-CH_2_CH_2_CH_2_-Si≡(OC_2_H_5_)_3_] = 14 cm^3^: 1.7 cm^3^.

The thermal stability of MCM-41 can be significantly improved by employing a low molar ratio of surfactant to tetraethyl orthosilicate (TEOS) during hydrothermal synthesis [35]. These MCM-41 silica adsorbents were synthesized conventionally [2,5,19,20,22,23,32]. The components were introduced into the Erlenmeyer flask and stirred for 1 h. The reaction mixture was then loaded into an autoclave and placed in a drying oven at 373 K for five days. The obtained product was filtered off, washed with redistilled water, and extracted with a mixture of 15 g of 36% HCl and 450 cm^3^ of ethanol at 323 K for about 6 h. The precipitate was filtered off and dried at 333 K. The mixtures were then gently cooled to room temperature and subsequently filtered and washed with triple deionized water several times, finally with a portion of isopropanol. After those operations, obtained in the above defined ways, samples were extracted in refluxed cycles for 6 h in aqueous alcohol with concentrated hydrochloric acid. The washed samples were gently dried for 5 h in an oven at 373 K. We have an intact (i.e., pristine or unmodified) adsorbent that can be lined with covalently bound guest phases.

It should be noted that TEOS can quickly become silicon dioxide after adding water during the synthesis stage of the intact MCM-41 adsorbent, also taking into account all subsequent features of modifying the pristine MCM-41 [36,37]:

Si(OC_2_H_5_)_4_ + 2 H_2_O → SiO_2_ + 4 C_2_H_5_OH.

However, in reality, the silica produced might be hydrated. This hydrolysis reaction is an example of a sol-gel process; the side product was ethanol. The reaction proceeds via a series of condensation reactions that convert the TEOS molecule into a mineral-like solid via the formation of 
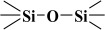
 linkages. At elevated temperatures (usually higher than 330 K), TEOS converts to silicon dioxide, and the volatile byproduct is diethyl ether [38,39]:

Si(OC_2_H_5_)_4_ → SiO_2_ + 2 (C_2_H_5_)_2_O

### 2.2. Investigation Methods

The pristine and surface-modified adsorbents were examined using X-ray diffraction (XRD), X-ray photoelectron spectroscopy (XPS), and N_2_-physisorption. A mathematical model of the average crystallite and crystalline powder involving the mean size and shape of the crystallite and mesopores (mesotunnels) inside was proposed. The crystalline microstructure of each sample was described by determining characteristic parameters of the model from experimental data, which were then explained backwards using the model. Both the siliceous frame and the organic lining were taken into account in the modelling. For these reasons, the complete characterisation of the chemical state of silica lined with n-propylotriethoxysilene, 3-aminopropyltriethoxysilane, and 3-mercaptopropyltriethoxysilane requires detailed knowledge of the nature of siloxane attachment to the mesopore surface, that is, the result of pristine silylation of the surface of MCM-41, as well as the nature of interactions, if any, between the amino group and other acid or base sites in the system [40,41].

## 3. Results of Experiments and Modelling

### 3.1. Measurements and Analysis of Nitrogen Physisorption Isotherms

**Figure 1:**
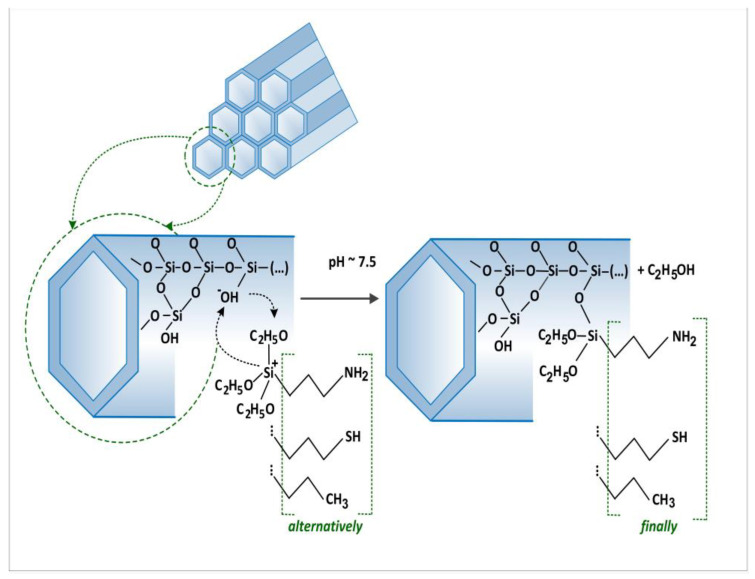
Methods of synthesis and modifications of the surface of the MCM-41.

We synthesised the siliceous material in terms of the framework order established with a surfactant with sixteen carbon atoms in alkyl chains, MCM-41-C16, as described in the introduction [42]. The methods of synthesis and modification of the surface of our MCM-41 are presented diagrammatically in Figure 1.

**Figure 2:**
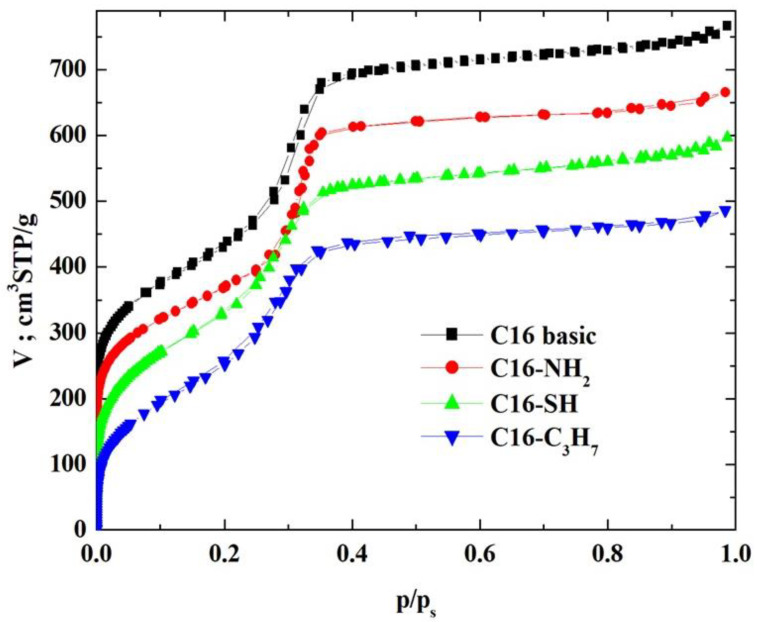
The N_2_-physisorption isotherms on the tested MCM-41.

N_2_ physisorption measurements on these MCM-41-C16 samples, both pristine and surface-modified (that is, lined with covalently bound guest phases), were performed employing the ASAP-2010 adsorption analyser from Micromeritics (Norcross, GA, USA). These adsorption isotherms were determined at 77 K within the relative pressure range from about 4.5 × 10^−6^ to 0.986, employing N_2_ of purity of 99.998% (which belongs to the borderline bases [43,44,45]). Before adsorption measurements, samples of the unmodified and modified silica adsorbents were pre-outgassed at 313 K for 24 h. The N_2_–physisorption isotherms for the pristine unmodified MCM-41 sample and the surface-modified samples are shown in Figure 2 in the linear scale of adsorption and relative pressure.

The N_2_-physisorption isotherms in the synthesised adsorbents (see Figure 2) were of type IV, which confirms the mesoporous nature of the investigated materials [2,21,46,47]. It is quite necessary to emphasise that Sing et al. [46] observed an excellent agreement between N_2_ and O_2_ adsorption on MCM-41s. However, different capillary condensation steps were observed for N_2_ at relative pressures of 0.05–0.06 and 0.15–0.25. The hysteresis loops were relatively narrow, suggesting that these adsorbents did not contain pores of various shapes, especially those adsorbents lined with organised monolayers of functional groups covalently bound to the mesoporous support, that is, 3-aminopropyltriethoxysilane, (-NH_2_), 3-mercaptopropyltriethoxysilane, (-SH) and *n*-propylotriethoxysilane, (-C_3_H_7_) groups. During the syntheses of these MCM-41s, we supposed that incorporating larger mesopores (or even mesotunnels [21,46]) into the siliceous adsorbent would be highly desirable if their surfaces were functionalised and employed to provide more surface functional groups, which has been confirmed by this analysis.

Type IV N_2_-physisorption isotherms are generally characteristic of mesoporous adsorbents [2,21,46]. The enrichment of N_2_ molecules in mesopores is determined by interactions of adsorbent (that is, MCM-41-C16 in our case) with adsorptive (that is, N_2_ molecules employed, which, according to the HSAB principle, belong to the borderline bases [43,44,45]). In the case of Type IV isotherms, the initial monolayer of the multilayer with increased concentration of N_2_ molecules in the walls of the mesopores (or even the mesotunnels), which, according to Thommes et al., takes the same path as the corresponding part of a Type II isotherm, is followed by pore condensation. It is consistent with two distinctive features of the Type H3 hysteresis loop, namely [21]:

(a) the N_2_-physisorption branch resembles a Type II isotherm;
(b) the lower limit of the N_2_ desorption branch, which, according to Thommes et al. [21], is typically located at $\frac{p}{p_{s}}$, is caused by cavitation.

Although many models were proposed to describe these adsorption phenomena, it was relatively easy to verify their accuracy by employing experimental N_2_-physisorption isotherms. This issue will be examined in more detail later in this article.

Three reference silica adsorbents, LiChrospher Si-1000 [48], Nucleosil 1000 [49], and Fransil-I [50], were employed for calculating the profiles of the α_s_-plots for all specimens in the absence of the reference materials lined with the following groups, i.e., n-propyl, aminopropyl, and mercaptopropyl. The suitable characteristics are collated in Table 1.

The total adsorbent-specific surface area, S_t_, was determined based on the slope of the straight line at the beginning of the α_s_ graph: $V\;\left[\mathrm{STP}\;cm^{3}g^{-1}\right]=V_{\mathrm{mi}}+\eta_{1}\alpha_{s}$ for 0.06 < α_s_ < 0.6. The slope of the line, ŋ_1_, is related to the total surface area of the MCM-41-C16 via the following relationship [49,51,52]:

(1) $$S_{t}=\frac{\eta_{1}S_{\mathrm{BET}}^{\mathrm{ref}}}{V_{0.4}^{\mathrm{ref}}}$$

where $S_{\mathrm{BET}}^{\mathrm{ref}}$ is the specific surface area of non-porous standard silica; $V_{0.4}^{\mathrm{ref}}$ is the adsorption volume of N_2_ on a reference adsorbent at 77 K.

The specific volumes of primary mesopores, V_me_, and the outer specific surface area, S_e_, were calculated based on the slope of the linear part of the α_s_ graph:$V\;\left[\mathrm{STP}\;cm^{3}g^{-1}\right]=V_{\mathrm{me}}+\eta_{2}\alpha_{s}$ for 1.25 < α_s_ < 2.4, for relative pressure more significant than the N_2_ condensation pressure in primary mesopores, i.e., for capillary condensation pressure in secondary mesopores. The slope of the line, ŋ_2_, is related to the external specific surface area of pristine MCM-41 through the following relationship [49,51,52]:

(2) $$S_{e}=\frac{\eta_{2}S_{\mathrm{BET}}^{\mathrm{ref}}}{V_{0.4}^{\mathrm{ref}}}$$

The S_BET_ specific surface area values [49,51,52] of the tested samples were determined based on low-temperature N_2_-physisorption. To estimate the values of the areas, the Brunauer–Emmett–Teller equation (BET) was employed in the following form:

(3) $$\frac{p}{p_{s}}{\left[V\left(1\;-\frac{p}{p_{s}}\right)\right]}^{-1}+\frac{C_{\mathrm{BET}}-1}{V_{m}xC_{\mathrm{BET}}}\frac{p}{p_{s}}$$

where: 

V—adsorption volume for a given relative pressure $\frac{p}{p_{s}}$;

p_s_—saturated vapor pressure of N_2_ at the measurement temperature, T;

V_m_—monolayer adsorption volume;

C_BET_—constant dependent on the adsorption energy and adsorption temperature (characterizing the adsorbate–adsorbent interaction).

The knowledge of monolayer adsorption volume, V_m_, allows us to determine the values of the specific surface area, S_BET_, if the surface occupied by the adsorbate molecule in the monolayer at the measurement temperature, T, is known, the so-called sitting area $\omega_{N_{2}}=0.1627\;{\mathrm{nm}}^{2}$, and molar volume of the adsorbate at the measurement temperature, $\vartheta_{N_{2}}=0.03468\;{\mathrm{cm}}^{3}{\mathrm{nm}\mathrm{ol}}^{-1}:$

(4) $$S_{\mathrm{BET}}=602.3\frac{\omega_{N_{2}}}{\vartheta_{N_{2}}}V_{m}\left[m^{2}g^{-1}\right]$$

For MCM-41 adsorbents, the process of adsorbate condensation inside the mesopores occurs at relative pressures located in the range in which the BET equation is used to describe the adsorption isotherm. The authors of the equation suggested a range of relative pressures ranging from 0.05 to 0.35. Therefore, to omit the capillary condensation range in the description with the BET equation, the relative pressure range in which the equation is exploited should be adjusted appropriately. This range, of course, depends on the size of MCM-41 mesopores, and as suggested by Sayari et al. [51], it varies from 0.04 to 0.06 to 0.10 to 0.25.

As mentioned earlier, the isotherms contain a jump in relative pressure of about 0.3, which reflects the capillary condensation of liquid nitrogen in homogeneous mesopores. It is somewhat less steep for the modified adsorbent. This is due to a slightly different interaction of N_2_ molecules with the outermost layer of its atoms forming silanol, 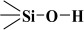, *n*-propyl, 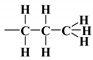, 3-mercaptopropyl, 
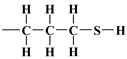
, and 3-aminopropyl groups 
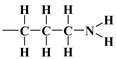
, compared to all interactions of N_2_ molecules with the outermost layer of unmodified adsorbent atoms, which contains only silanol groups. After a condensation jump, the isotherm profiles include a long plateau, indicating that the mesoporous structure of the tested adsorbents consists mainly of primary mesopores.

### 3.2. XPS Characterization of the Absorbents Tested

To correctly interpret the adsorption results of N_2_ molecules in complex structures of MCM-41 (both pristine and modified) presented in the previous subsection, we also want to determine the elemental composition of their outermost layers precisely. Thus, the powder samples for the XPS analyses were pressed into pellets using a hydraulic press. After being degassed in a load lock for 16 h, they were transferred to the analysis chamber of the Prevac UHV system, which was equipped with a system to neutralise the surface charge (Rogów, Poland).

All measurements were performed using the Scienta R4000 (Osolin, Poland) analyzer equipped with a monochromatic source (XM 650, 0.2 eV band) Al K_α_ source (SAX-100, 1486.6 eV, 10 mA, 12 kV). The base pressure of the instrument was 5 × 10^−9^ mbar. The Casa XPS version 2.3.19 PR1 software was used to process the XPS spectra and calculate the results.

**Figure 3:**
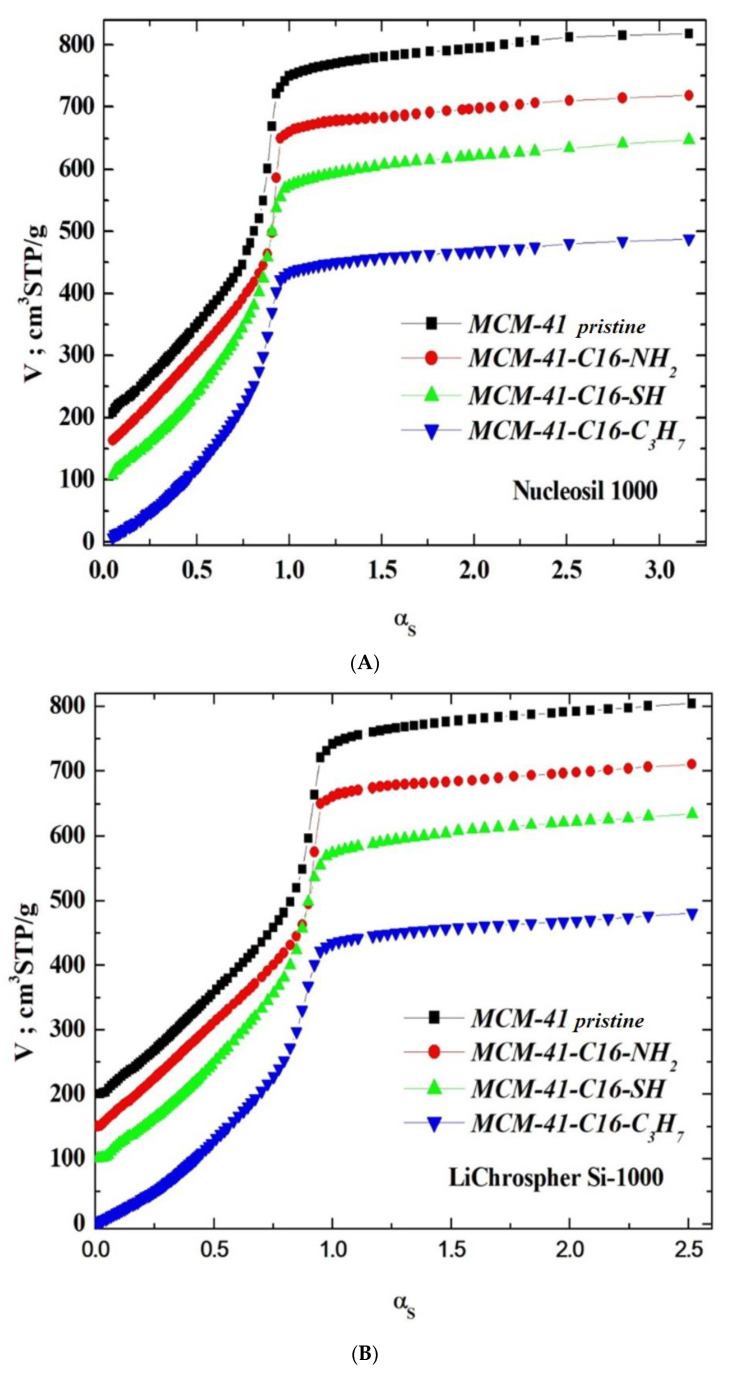

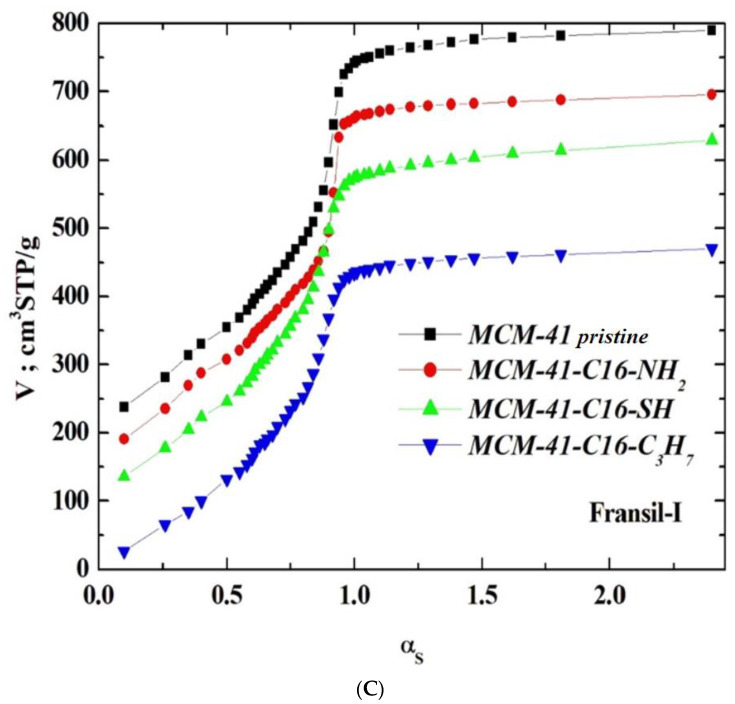
(**A**). The $\alpha_{S}$-plots, computed with respect to the sstandardisedsilica Nucleosil 1000. The plots for the pristine samples of MCM-41-SH, MCM-41-NH_2_, MCM-41 are shifted by 100, 150, and 200 STP cm^3^ g^−1^, respectively. (**B**). The $\alpha_{S}$-plots, computed with respect to the sstandardisedsilica LiChrospher Si-1000. The plots for the pristine samples of MCM-41-SH, MCM-41-NH_2_ are shifted by 100, 150, and 200 STP cm^3^ g^−1^, respectively. (**C**). The $\alpha_{S}$-plots, computed with respect to the standardised silica Fransil-I. The plots for the pristine samples of MCM-41-SH, MCM-41-NH_2_ are shifted by 100, 150, and 200 STP cm^3^ g^−1^, respectively.

**Figure 4:**
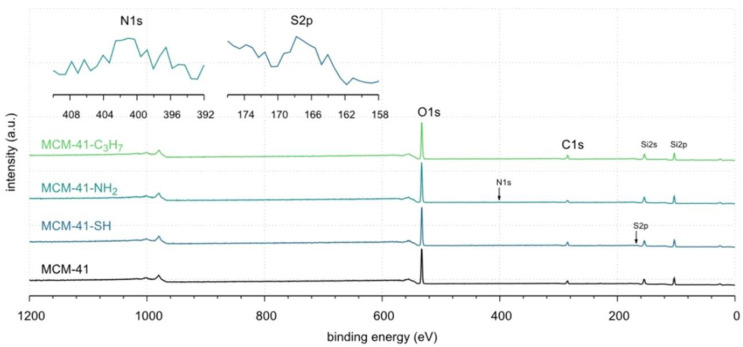
The XPS survey spectra of MCM-41: pristine and lined with organised monolayers of functional molecules: MCM-41, MCM-41-SH, MCM-41-NH_2_, and MCM-41-C_3_H_7_, respectively.

The XPS spectra of the samples tested are shown in Figure 3. All spectra contain two strong peaks derived from silicon at ~103.3 eV and oxygen at ~533.1 eV. Three low-intensity peaks are attributed to carbon at ~284.8 eV, nitrogen at ~401.1 eV, and sulfur at ~167.8 eV (small tails inside the main plot). Details of surface composition are summarised in Table 2. The full survey spectra of our study are depicted in Figures S1–S4 (in Supplementary Materials). However, the surface concentrations of nitrogen and sulfur are low and amount to 1.0% at and 1.6% at, respectively (see Table 2). The data clearly show that the molecules corresponding to the functional groups of interest (i.e., -NH_2_ and -SH) were covalently bound to the pristine surface of MCM-41 [40]. Unfortunately, these surfaces lined with organised monolayers of functional molecules, -C_3_H_7_, -C_3_H_6_-NH_2_, -C_3_H_6_-SH groups, could not be clearly distinguished from adventitious carbon (see Figure 4, Table 2, and Figures S1–S5 in Supplementary Materials) [53]. According to the XPS results, the covalently attached monolayers (CAMs) of monofunctional silanes, HS-CH_2_CH_2_CH_2_-Si≡(OC_2_H_5_)_3_, H_2_N-CH_2_CH_2_CH_2_-Si≡(OC_2_H_5_)_3_, CH_3_CH_2_CH_2_-Si≡(OC_2_H_5_)_3_, were anchored to the silica surface [40]. Notwithstanding these facts, the thickness of Si-supported CAMs indicated a complete modification of the surface along with the formation of hydrophobic CAMs of *n*-propyl and hydrophilic aminopropyl and mercaptopropyl groups [54,55].

In this case, an increase in carbon concentration from 13.5% at. to 17.1% at. can only serve as a qualitative indicator confirming the modification of the surface with covalently bound groups containing -C_3_H_7_ and -C_3_H_6_-chains in their structures.

Therefore, a distinction cannot be made between an adventitious carbon (AdC) that is incompletely washed away from the surface of MCM-41 [53] by creating C_2_H_5_OH and (C_2_H_5_)_2_O, which can be thought of as composed of the ethyl carbonium ion, C_2_H_5_^+^, and the hydroxide ion, OH^−^, or the base ethoxide ion, C_2_H_5_O^−^, and its derivatives or the carbon bound in ethyl groups belonging to the groups *n*-propyl, 3-aminopropyl, and 3-mercaptopropyl triethoxysilane [36,37,38,39,44]. An AdC is generally considered to be a thin overlayer of mainly hydrocarbon material (attributed to 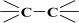 and 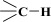 of aliphatic carbons; see Section 3.1) that accumulates on silica surfaces [36,37,38,39]. In other words, the carbon bound in the ethylene groups could have been obscured by the carbon not washed from the surface of MCM-41 and its derivatives. Unfortunately, the observed AdC is a significant disadvantage of the MCM-41 extraction synthesis method, as a considerable amount of AdC also occurs on the surface of the pristine MCM-41 adsorbent.

The high-resolution spectra of O1s, Si2p, and C1s are shown in Figure 4. They show no changes that could be caused by the immobilisation of n-propyl, mercaptopropyl, or aminopropyl groups.

The fitted O1s and Si2p spectra are shown in (Supplementary Materials) Figures S6 and S7, respectively. Binding energies of the most intense O1s peaks at ~533.0 eV indicate that they can be assigned to oxygen-silica bridges (
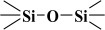
in the tetrahedral configurations of siloxanes in pristine MCM-41s (see in Supplementary Materials, Figure S6). The O1s spectra for modified MCM-41 reveal an increased share of the second peak with a binding energy of ~533.8 eV (see in Supplementary Materials Table S2), which is attributed to siloxane groups (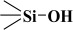) [56,57] present on the surface of the tested adsorbents and carbon by random species with a single oxygen bond [53,58,59,60]. This increase is probably due to the remaining water and organic compounds employed in the synthesis processes. The smallest remaining peak at ~531.3 eV was assigned to unusual and unavoidable quantities of double carbon binding with oxygen. Furthermore, the fitted spectra of the C1s [53,61,62] and Si2p [56,57,63,64] spectra are shown in Figures S5 and S7.

### 3.3. XRD Characterization of the Absorbents Tested

**Figure 5:**
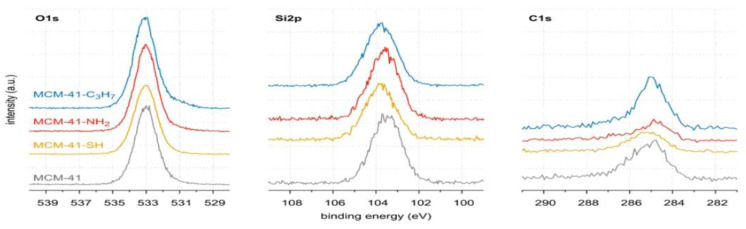
The XPS high-resolution O1s, Si2p, and C1s spectra of pristine MCM-41 and lined with organized monolayers of functional molecules. Details are given in (Supplementary Materials) Figures S5–S7.

The powder XRD patterns for all MCM-41 samples were recorded on a Panalytical EMPYREAN (Houston, TX, USA) diffractometer (working at a voltage of 40 kV and a current of 25 mA, with a divergence slit of ${\left(\frac{1}{32}\right)}^{{}^{\circ}}$ and receiving slit of 0.05 mm), equipped with an area detector working in scanning line detector mode, using Cu Kα radiation at diffraction (2*θ*) angles from 0.9° to 8.0° with step of 0.05° (for samples of pristine MCM-41 and covered with propyl derivatives). The XRD patterns were analysed to determine Bragg angles as positions of peak maxima. First, for each pattern, the background was determined as a monotonically decreasing smoothed natural cubic spline function to approximate together the manually selected fragments of the pattern, and then the background was subtracted (as illustrated in Figure 5).

**Figure 6:**
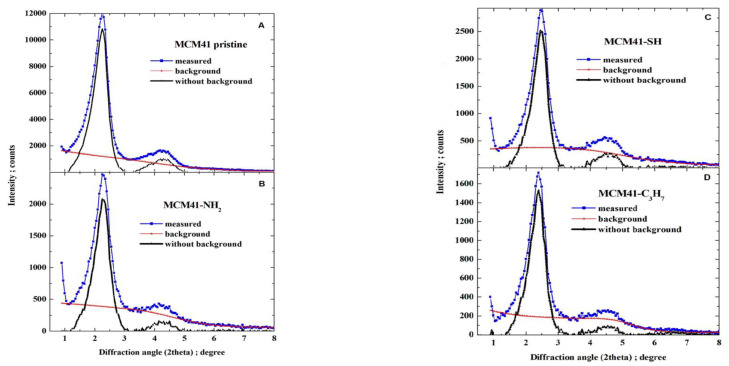
(**A**) Pristine MCM-41, and (**B**–**D**) MCM-41 silica lined with organised monolayers of functional molecules covalently bound to the mesoporous support—XRD diffraction patterns.

After subtracting the background, each pattern was represented with the small step of diffraction angle as a natural cubic spline by applying optimal smoothing [65] with an error estimate derived from quantum count statistics of X-ray intensity measurements [66,67,68]. The optimal smoothing was performed so that the root-mean-square difference between the vector of measured background-corrected X-ray intensities and the vector of corresponding smoothing spline was equal to the root-mean-square estimate of the error in the experimental pattern. Finally, the position of the maxima was determined by finding the local maxima. Six peaks were observed that could be indexed in accordance with the hexagonal crystalline lattice of the constant a, as corresponding to reflections: 100 (strong), 110 (weak), 200 (weak), 210 (very weak), 300 (very weak), and 220 (extremely weak), as illustrated in Figure 6.

The axial divergence of the X-ray beam significantly affected the peak shapes (i.e., X-ray diffraction peak profiles). This effect is unavoidable at such small diffraction angles and causes asymmetric broadening of the line profiles from the side of smaller diffraction angles, especially in peaks at 100. The shifts of the maximum peak positions were so small that they could be neglected in further calculations. The Cu Kα_1_ wavelength was exploited to determine the unit cell parameter. Both the crystalline lattice constant (the unit cell parameter a) and the error estimate were determined from all six peak positions to minimise the relative error calculated as the square root of the mean squares of relative errors of reproducing the Bragg angles by experimental peak maximum positions. The absolute error ∆a [nm] in the unit cell constant a [nm] was then estimated by this relative error estimate as the product of its value, ∆a = a × e_r_. The results are shown in Table 3.

The interplanar spacings $d_{100}$ [nm] with error estimates were recalculated from the values of a. Due to small differences between the unit cell constant for all samples (produced in similar ways), being significantly inferior to error estimates e_r_ (and similar processes of synthesis MCM-41 for all samples), the same unit cell constant, calculated as the arithmetic mean of four preliminary values, equal to a=4.498 nm, was taken as representative for the four samples (see last row of Table 4) and the error estimate was recalculated for data combined from all samples; the Bragg diffraction angles corresponding to six analysed reflections 100, 110, 200, 210, 300, 220 were then 2.266, 3.926, 4.533, 5.998, 6.802, 7.856 degrees.

**Figure 7:**
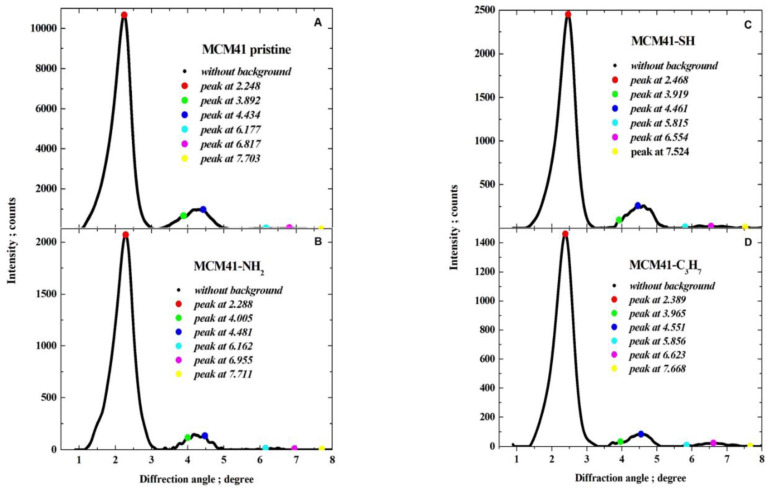
Smoothed background-corrected XRD diffraction patterns (λ=0.15406 nm) from (**A**) pristine MCM-41 silica and MCM-41 silica lined with organised monolayers of functional molecules (**B**) (-C_3_H_6_-NH_2_), (**C**) (-C_3_H_6_-SH), (**D**) (-C_3_H_6_-C_3_H_7_) covalently bound to the mesoporous support, depicting marked peak maxima (for reflections 100, 110, 200, 210, 300, 220).

Smoothed background-corrected XRD diffraction patterns are shown in Figure 7.

For further computations and modelling, this average value of unit cell constant was taken as characterizing the silica MCM-41 crystalline frame in all samples.

### 3.4. Mathematical Model of the Crystalline Microstructure

**Figure 8:**
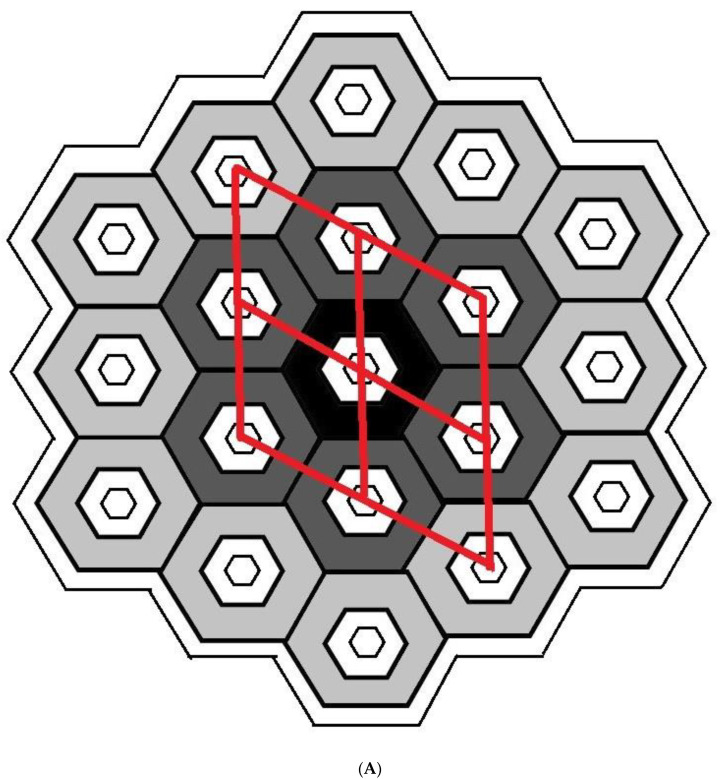

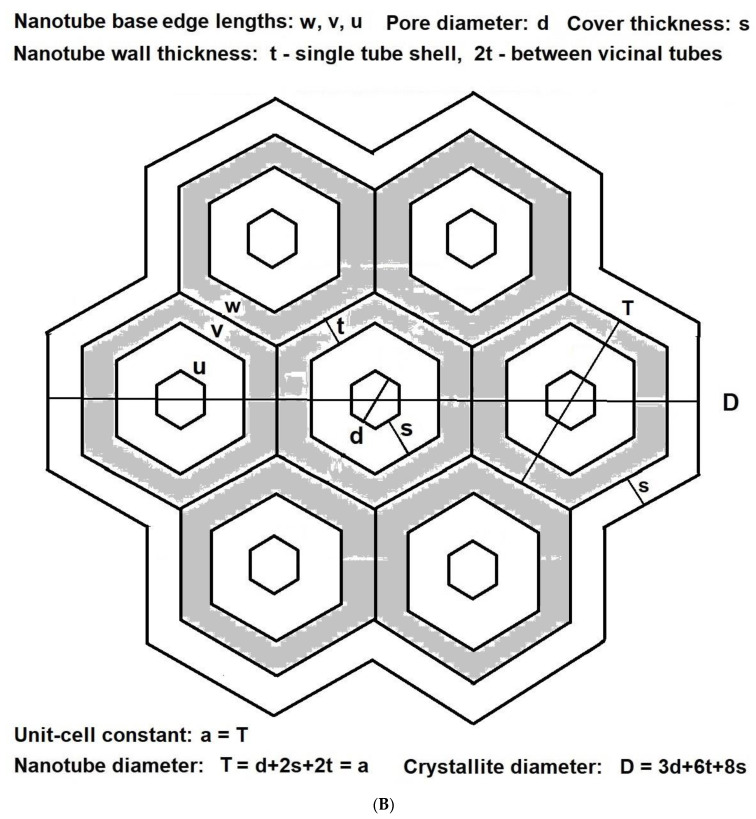
(**A**) Definition of mean crystallite: base (or cross-section) of a single silica crystallite, assumed to be a monocrystalline grain of the shape of a prism comprising rings of hexagonal nanotubes unified into a honeycomb structure around the central tube (two differently coloured rings are shown, and four vicinal unit cells are sketched), covered with an amorphous layer (white). (**B**) Basic parameters of crystallite base for one-ring crystallite with silica shell walls (grey) lined with another amorphous material (white).

The characteristics of all tested adsorbents were preliminarily calculated from XRD diffraction patterns and the N_2_–physisorption isotherms. A mathematical model of the crystalline microstructure was then proposed to explain in more detail. Although simplified, it enabled the computation of the approximate microstructural characteristics of investigated crystalline powders, imitating well the experimentally determined ones and explaining correlations between microscopic and macroscopic properties. Adsorbents like MCM-41 or SBA15 feature nanotubes aligned to the crystal’s six-fold rotational axis. Let us consider a monodisperse set of the same monocrystalline grains (single crystallites) of prismatic shape, each consisting of tightly connected identical hexagonal nanotubes of the same length and hexagonal bases with hexagonal holes inside (with both base hexagons of corresponding sides parallel, as sketched in Figure 8A,B).

Let such a crystallite contain a central tube (k=0) with subsequent rings (k=1,2,3,⋯) of similar tubes with hexagonal mesopores (Figure 8A), all of height H. The external and internal faces of each nanotube are rectangles of edges w, H or u, H, where w is the base outer edge length related to the unit cell constant as $a=\sqrt{3}\times w$, and u is the base inner edge length of the base. The characteristics of a single crystallite consisting of k tube rings, and a whole monodisperse set of such crystallites can then be expressed as functions of these parameters (Figure 8B):

- the short mesopore diameter (distance between opposite parallel rectangular faces of a mesopore or opposite internal nanotube base sides) as $d=\sqrt{3}\times u$, the long mesopore diameter (distance between opposing parallel nanotube edges or corresponding internal base vertices) as 2×u, mesopore wall thickness (in the direction perpendicular to the face) as $t=\frac{1}{2}\times \sqrt{3\;}\times (w\;-\;u)$, the inter-mesopore wall thickness (in the direction perpendicular to the face) as $\sqrt{3}\;\times (w\;-u)$, the diameter of crystallite base (the largest distance between opposite tube side faces) as $D=\left(2k+1\right)\times \sqrt{3\;}\times w$;
- the surface area of the crystallite base (with the total area of internal mesopore sections excluded) as $\frac{3}{2}\times \sqrt{3\;}\times (3\times k\times \left(k+1\right)+1)\times \left(w^{2}-\;u^{2}\right)$, the surface area of internal crystallite mesopore faces as $6\times (3\times k\times \left(k+1\right)+1)\times u\times H$, the surface area of crystallite external side faces (perpendicular to base) as 6×(2×k+1)×w×H;
- the total volume of the whole crystallite (solid with internal mesopores) as $\frac{3}{2}\times \sqrt{3\;}\times (3\times k\times \left(k+1\right)+1)\times w^{2}\times H$, the total volume of crystallite mesopores (nanotube internals) as $\frac{3}{2}\times \sqrt{3\;}\times (3\times k\times \left(k+1\right)+1)\times u^{2}\times H$, the total volume of crystallite walls (bulk material of honeycomb structure) as $\frac{3}{2}\times \sqrt{3\;}\times \left(3\times k\times \left(k+1\right)+1\right)\times {(w}^{2}-u^{2})\times H$.

Any specific surface area for the whole powder sample (as a set of such crystallites) can be consequently calculated as a quotient of the correspondent crystallite surface area divided by the product of the volume of crystallite walls multiplied by the density of material constituting walls of the honeycomb structure. Additionally, to obtain these characteristics computed for the polycrystalline powder model to be compatible with those calculated from the N_2_–physisorption isotherms, a factor such as surface roughness, r, shall be introduced. It might be interpreted as the quotient of the surface area of crystallite walls lined with a monolayer of nitrogen molecules divided by the surface area calculated for planar geometrical faces. All formulae can be adopted to describe silica lined with organised monolayers of functional molecules covalently bound to the mesoporous support (as illustrated in Figure 8A,B).

A similar model of porous silica microstructure was considered, e.g., in [51,52,69] (although different in many details).

### 3.5. Results of the Computations and Discussion

The mathematical model of crystallite and polycrystalline powder was exploited to explain the adsorptive characteristics of the specimens under study concerning crystalline structure and microstructure. The crystallite parameters of the model—the number of nanotube rings k, height H, edge length u (Figure 7B), and surface roughness r—were determined to achieve minimal differences between the specific mesopore volume $\bar{V_{p}}$, total specific surface area $\bar{S_{t}}$, pore-specific surface area $\bar{S_{p}}$, and external specific surface area $\bar{S_{e}}$, computed from experimental nitrogen adsorption isotherm (single bar-marked in Table 4) and those calculated for the model (double bar-marked). The corresponding inverse problem is unstable and, therefore, it was solved through minimization of the regularizing functional being the sum of similarity functional e and stabilizing functional f; the similarity functional was defined as the root-mean-square relative error:

(5) $${e=e\left(a,\rho ;k,H,u,r\right)=\left\{\frac{1}{4}\left[{\left(\frac{\bar{V_{p}}-\overset{=}{V_{p}}}{\bar{V_{p}}}\right)}^{2}+{\left(\frac{\bar{S_{t}}-\overset{=}{S_{t}}}{\bar{S_{t}}}\right)}^{2}+{\left(\frac{\bar{S_{p}}-\overset{=}{S_{p}}}{\bar{S_{p}}}\right)}^{2}+{\left(\frac{\bar{S_{e}}-\overset{=}{S_{e}}}{\bar{S_{e}}}\right)}^{2}\right]\right\}}^{\frac{1}{2}}$$

with the assumed density of crystallite wall material (nanotube wall material) ρ and unit-cell parameter a (or w, the external edge length of hexagonal base of a single nanotube hexagonal base; $w=3^{-\frac{1}{2}}a$); the stabilizing functional was taken as quadratic functional.

(6) $$f\left(w;k,H,u,r\right)=\alpha \left({\left(u-\;w\right)}^{2}+{\left(\frac{H}{D}\right)}^{2}+k^{2}+r^{2}\right)$$

with regularization parameter α>0. For each sample, two rounds of computations were performed. In the first round, $\overset{=}{k},\overset{=}{H},\overset{=}{u},\overset{=}{r}$, and $\overset{=}{\alpha }$ were found as minimizing functional e+f:

(7) $$\left(\overset{=}{\alpha },\overset{=}{k},\overset{=}{H},\overset{=}{u},\overset{=}{r}\right)=\arg\min\left\{\left(e+f\right)\left(a,\rho ;k,H,u,r\right):\;\alpha ,k,H,u,r>0;\;e\left(a,\rho ;k,H,u,r\right)=\overset{=}{e}\right\}$$

with the regularisation parameter chosen according to the discrepancy principle [70,71] when the resulting relative error is approximately equal to an estimate from the experimental data (last column in Table 4). In the second round, the number of rings k was rounded to the nearest integer and fixed and then the calculations were performed with three independent variables H,u,r and α (with the stabilising functional reduced to three quadratic terms). First, the bare silica sample MCM-41 was analyzed; the bulk density of the silica shell (nanotube wall material) was assumed equal to $2.6487\;g{cm}^{-3}$, i.e., the density of α-quartz [72,73], which is a basic allomorph of silica with a hexagonal crystalline structure. Then, the resulting nanotube base inner edge length u (as well as base outer edge length w) was assumed to be the same for the silica shell in other samples lined with propyl derivatives (as the base medium edge length v). The crystallite model was extended to account for organized amorphous monolayers of functional molecules covalently bound to the mesoporous support of *n*-propyl, 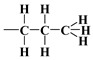, 3-aminopropyl [40], 
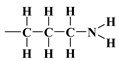
 or 3-mercaptopropyl, 
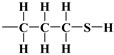
, lining crystalline silica shell inside pores and outside the crystallites (Figure 7). The density of the cover layers was assumed to be equal to $0.719\;g{cm}^{-3}$, i.e., the density of 3-aminopropyl under standard conditions, taken as representative for these three substances. The calculations were repeated three times in the same way, with four parameters $w,v,\rho_{s},\rho_{c}$ ($\rho_{s}$, $\rho_{c}$ being the densities of crystalline shell and the alkyl cover):

(8) $$\left(\overset{=}{\alpha },\overset{=}{k},\overset{=}{H},\overset{=}{u},\overset{=}{r}\right)=\arg\min\left\{\left(e+f\right)\left(w,v,\rho_{s},\rho_{c};k,H,u,r\right):\;\alpha ,k,H,u,r>0;\;e\left(a,\rho ;k,H,u,r\right)=\overset{=}{e}\right\}$$

The relative error level $\overset{=}{e}$ (of 0.01) for computing the regularization parameter was chosen as representative for all samples, a little higher than the root-mean-square value from estimates $\bar{e}$ computed from a formula similar to (5) while accounting only for ${\overset{-}{V}}_{p},\;{\overset{-}{S}}_{t},\;{\overset{-}{S}}_{p}$ and corresponding values, as presented in Table 1.

It was found from numerical experiments that it is impossible to calculate the density of the silica shell and other parameters reliably (as presented in Table 5) even when regularization is applied. Therefore, both densities of the silica shell and different guest phases were assumed as known parameters. The results of the measurements, modelling, and computations are collected in Table 4 (adsorption characteristics) and Table 5 (microstructural characteristics). In Table 4 are presented the adsorption characteristics of pristine MCM-41 adsorbents or lined with organized monolayers of functional molecules covalently bound to the mesoporous support: n-propyl or aminopropyl, or mercaptopropyl (means from the results of measurements with three reference adsorbents, from Table 1), with mesopores accounted for (the single bar denotes the experimental values). Headings in Table 4 indicate the following:

$\bar{V_{p}}$ [${\mathrm{cm}}^{3}g^{-1}$]—specific volume of mesopores;

$\bar{S_{t}}$ [$m^{2}g^{-1}$]—the total specific surface area of crystalline grains;

$\bar{S_{e}}$ [$m^{2}g^{-1}$]—the external specific surface area of crystalline grains;

$\bar{S_{p}}$ [$m^{2}g^{-1}$]—the surface area of mesopores in monocrystalline grains;

${\overset{-}{S}}_{p}={\overset{-}{S}}_{t}-\;{\overset{-}{S}}_{e}$ (all calculated from *α*_s_-plot);

S_BET_—the specific surface area of crystalline grains (determined by the Brunauer–Emmett–Teller method).

The double bar denotes the corresponding values computed for the model polycrystalline powder with microstructure parameters specified from the experimental specimen characteristics.

$\overset{-}{e}$ indicates the root-mean-square relative error of the root-mean-square of approximating experimental data (mesopore specific volume $V_{p}$ and two specific surface areas, S_t_, S_e_) compared to their mean values.

$\overset{=}{e}$ indicates the root-mean-square relative error of the root-mean-square of approximating the averaged experimental characteristics by the model characteristics (the same relative error chosen as representative of the experimental data was achieved by applying the regularisation method with a proper value of the regularisation parameter); an excessive decimal digit is left differences to illustrate the differences better.

In Table 5 are presented the characteristics of four samples of MCM-41, bare or lined with organized monolayers of functional molecules covalently bound to the mesoporous support, n-propyl, aminopropyl, and mercaptopropyl, computed from the results of measurements using nitrogen adsorption and XRD (letter symbols with single bar) or calculated by employing the model of monocrystalline grain with parameters determined from these experimental data (letter symbols with double bar). Headings in Table 5 indicate the following: $\bar{a}[nm]$—the unit cell constant, $\bar{w}$ $\left[nm\right]$—the external edge length of the hexagonal base of the component nanotube ($\bar{a}=\sqrt{3}\times \bar{w}$), $\hat{w}$ [nm]—the internal edge length of the hexagonal base of silica shell of the component nanotube, $\overset{=}{w}$ [nm]—the internal edge length of the hexagonal base of a component nanotube (with cover), $\overset{=}{\rho }$ $\left[cm^{3}g^{-1}\right]$—the mean density of nanotube walls in crystallites with cover (between 2.6487 for α-quartz shell and 0.719 for aminopropyl cover), $\overset{=}{d}=\sqrt{3}\times \overset{=}{w}$ [nm]—pore diameter, ${\overset{=}{t}}_{e}$ [nm]—the thickness of external silica shell of nanotube, ${\overset{=}{t}}_{i}$ [nm]—the thickness of propyl or aminopropyl or mercaptopropyl cover inside nanotubes and outside crystallite [nm] ($\frac{1}{2}\sqrt{3}\times \left(\bar{w}-\overset{=}{w}\right)=\frac{1}{2}\sqrt{3}x\left(\left(\bar{w}-\hat{w})+(\hat{w}-\overset{=}{w}\right)\right)={\overset{=}{t}}_{e}+{\overset{=}{t}}_{i}$), $\overset{=}{L}$—the number of nanotube rings around the central one composing together the mean model crystallite, $\overset{=}{D}$ [nm]—the diameter of mean crystallite, $\overset{=}{H}$ [nm]—the height of mean crystallite, $\overset{=}{p}$—the mean porosity (contribution of whole mesopore volume to total crystallite volume, including mesopores), $\overset{=}{r}$—the surface roughness; one excessive decimal digit is preserved in all numbers to better display the differences between corresponding parameter values.

It should be emphasised that proton donors in hydrogen bonds can be, among others, the following end groups: hydroxyl (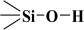), aminopropyl [40] (
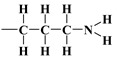
), and mercaptopropyl (
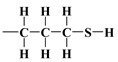
), considered in this work. One atom from the pair, nitrogen, oxygen, or sulfur (electron donor), is covalently bonded to the hydrogen atom, and electrons are shared unequally; its high electron affinity causes hydrogen to take a slight positive charge. For these reasons, the electrostatic force of attraction between a hydrogen atom (which is covalently bonded to a more electronegative ‘donor’ atom or group) and another electronegative atom that has a single pair of electrons (the hydrogen bond acceptor) should be carefully analysed. Therefore, considering the electron acceptor donor-acceptor (EDA) results in the previously cited paper by Grajek et al. [42] and Fryxell et al. [74], we conclude that forming a ‘bent-over posture’ for some surface-functionalized organosilanes is very likely. Therefore, the aminopropyl, –C_3_H_6_-NH_2_, and mercaptopropyl, -C_3_H_6_-SH, groups are attached by hydrogen bonding interactions between the amino and mercaptopropyl end groups and the surface silanols [75,76].

In this case, the surface properties of the tested siliceous adsorbents by nitrogen molecules may be rationalised in terms of the Hard and Soft Acids and Bases (HSAB) principle. Thus, according to the HSAB principle, the silanol,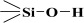, and aminopropyl [40], 
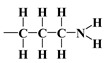
, groups are hard bases, and their electron donor atoms (i.e., O and N) have high electronegativity and low polarizability [43,44,45]. However, an amino group is a strong electron donor toward the 
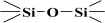
 bonds’ high reactivity such that the 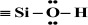 bonds’ polarities are increased [43,44,45]. As a consequence, there could be a notable attraction between –NH_2_ and OH groups from the surface through hydrogen bonding, leading to the bending of the organic linker. This results in a decrease in the number of available surface –OH groups for a reaction with an organosilicon compound and, finally, in a relatively moderate decrease in $\bar{V_{p}}$ of this material.

The mercaptopropyl groups, 
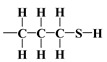
, are soft bases, and their electron donor (i.e., S) atoms have low electronegativity and high polarizability. Therefore, they are easy to oxidise and hold their valence electrons loosely. The presence of a soft donor in the -SH group should lead to less effective hydrogen bond formation with the –OH group containing the strongly electronegative and, hence, hard in terms of HSAB theory, oxygen atom. In summary, this will lead to an incompatible pairing as, according to HSAB theory, hard–hard and soft–soft interactions are the most efficient. This may finally lead to a situation where bending the organic linker containing the -SH group is less efficient. If so, then the amount of surface –OH groups prone to modification is higher than for the amino analogue, leading to the formation of a more densely covered surface and also decreasing the $\bar{V_{p}}$ value for this material.

The differences between lining layers observed here may also be the result of a lower coverage density, allowing for a wide-area lateral binding of ligands on the surface of the substrate. The chromatographically confirmed set of interactions would lead to a ‘bent posture’ for our surface-functionalized organosilanes. The CAMs of 
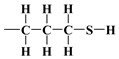
, 
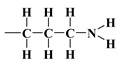
, and surface groups, 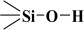, may behave as deposited on silica molecules with physical properties similar to liquids in which the effects of hydrogen bonding dominate the local structure [54,55,73]. Although the definition and characterisation of the hydrogen bonds is not straightforward, these hypotheses are given by the following results of macroscopic modelling of the crystalline microstructure for all investigated specimens.

**Figure 9:**
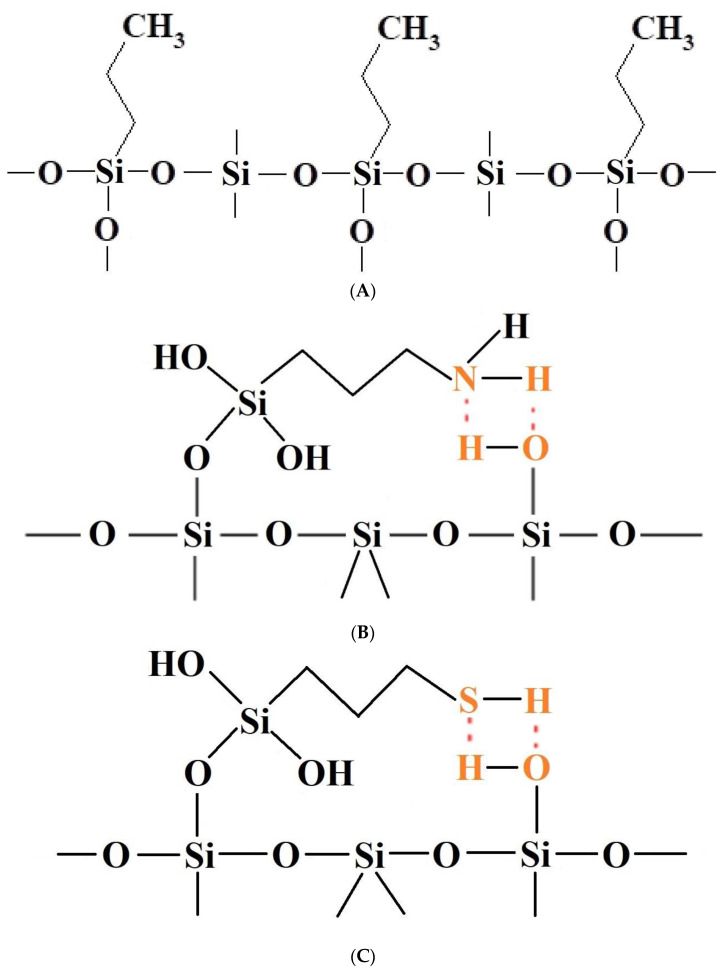
(**A**) Diagrammatic representation of the suggested arrangement of the silica surface lined with organised monolayers of functional molecules (-C_3_H_6_-C_3_H_7_) covalently bound to the mesoporous siliceous support, resulting in a ‘upright posture’ for our surface-functionalized organosilanes [74,75,76]. (**B**) Diagrammatic representation of the suggested arrangement of the silica surface lined with organised monolayers of functional molecules (-C_3_H_6_-NH_2_) covalently bound to the mesoporous siliceous support, which would result in a ‘bent over posture’ for our surface-functionalized organosilanes [74,75,76]. (**C**) Diagrammatic representation of the suggested arrangement of the silica surface lined with organised monolayers of functional molecules (-C_3_H_6_-SH) covalently bound to the mesoporous siliceous support, resulting in a ‘bent posture’ for our surface-functionalized organosilanes [74,75,76].

For samples with alkyl coatings, the layers’ thicknesses, organised monolayers of functional molecules covalently bound to the mesoporous support, are compatible with the possible structure of these layers on the silica surface (Figure 9). A minor surface roughness (0.91) was found for the bare silica sample. The 3-aminopropyl group is tightly connected on the silica surface at both ends, with a strong hydrogen bond of the amine group, resulting in the thinnest coating layer thickness (36 pm) and a slightly more significant surface roughness (0.95). In turn, the 3-mercaptopropyl group is similarly connected to the silica surface. However, the hydrogen bond with the 3-mercaptopropyl group is not as strong, and the sulfur atom is larger than the nitrogen atom, resulting in a greater thickness of the coating layer (84 pm) and a more significant surface roughness (0.99). On the other hand, the propyl group is connected only on one bond, resulting in the most significant coating layer thickness (135 pm) and the most considerable surface roughness (1.04). The appearance of the corresponding bonds in all four cases was confirmed from observations of the XPS analysis (Table 2); the characteristic bonds featured only specific samples. The thickness of alkyl coating on the surface of crystalline grains in each of the three specimens should be interpreted in the context of measurements of the specific surface area and mesopore volume using nitrogen adsorption. In the first synthesis stage, a silica shell is formed with a layer of the hydroxy group on the surface (both external and inside of the mesopores). In the process of lining with propyl derivatives, most of the hydroxy group is replaced by the ethoxy group and the alkyl derivative group (*n*-propyl or 3-aminopropyl or 3-mercaptopropyl), which results in only a slight change in surface area accessible for nitrogen (Table 1) and small thickness calculated for model cover layer, especially when the functional group is tightly connected to the surface silicon atoms on both ends, particularly 3-aminopropyl with a corresponding layer thickness of only 36 pm (to compare to the typical organic bond length of around 140 pm). The roughness should be interpreted as in the context of nitrogen N_2_ physisorption exploited as a measurement technique. When the moieties of N_2_ of length around 220 pm form a monolayer on the surface of the hexagonal section of silica mesotunnels with edge length around 2 nm, it is sometimes not possible to perfectly cover the entire area of rectangular wall surfaces with them (when these two sizes are incommensurate), especially at corners. Then, the roughness value (i.e., the quotient of surface area covered with nitrogen divided by geometric surface area of the idealised wall surface) becomes smaller than one. In the opposite case, when the surface roughness is low with respect to nitrogen molecules (as in the case of the n-propyl functional group bonded to the silica surface), the roughness value can be larger than one because the nitrogen monolayer can be slightly folded.

The outer sizes (diameter and height) of a mean crystallite are small in bare silica samples and more extensive in the rest of our studied silicas, which can be explained mainly as a result of differences in processes of synthesising, especially from the more significant time of processing and slightly higher temperatures applied to samples lined with monolayers of functional molecules (i.e., 3-aminopropyltriethoxysilane, (-NH_2_) [40], 3-mercaptopropyltriethoxysilane, (-SH), and *n*-propylotriethoxysilane (-C_3_H_7_) groups) covalently bound to the mesoporous siliceous material ordered with $SiO_{4}^{4-}$ tetrahedra.

## 4. Conclusions

Four samples of crystalline siliceous adsorbents of MCM-41 mesoporous frame were successfully synthesised as a primary material (pristine), and MCM-41 lined with organised monolayers of functional molecules covalently bound to the mesoporous support containing groups: 3-aminopropyltriethoxysilane or 3-mercaptopropyltriethoxysilane or *n*-propyltriethoxysilane. The results of the XPS research confirmed that the previously mentioned chemical groups were associated with a silica substrate as a characteristic chemical composition typical of that known in general chemistry. The contributions of atomic bonds [% at] in pristine MCM-41 and MCM-41 lined with organised monolayers of functional molecules clearly follow theoretical expectations. Furthermore, the number of these groups related to the MCM-41 surface and the results of modelling their arrangement on the pristine adsorbent surface may indicate the formation of hydrogen bonds between the -SH-HO- groups and the -NH_2_-HO- groups.

The formation of a hexagonal structure of porous crystalline silica material with a fundamental unit cell constant of around 4.5 nm was identified by analysis of an XRD pattern. A model of the crystalline microstructure as a monodisperse set of the same prismatic crystallites formed of hexagonal nanotubes was supposed and successfully used to explain the adsorption characteristic (specific surface areas and mesopore-specific volume) of the materials under study. The crystallites were considered as the same (monodisperse) monocrystalline grains in the form of prisms containing tightly connected nanotubes. In the frame of this model, the pore diameter of 3.7 nm was estimated in bare silica and slightly smaller (3.4 nm to 3.6 nm) in specimens with silica MCM-41 shell lined with propyl derivatives. The thickness of the coating monolayer was found to be compatible with the shape of an alkyl group and the bonding model. The roughness of the surface was calculated to be smaller than that in bare silica and two lined specimens and larger than that in the specimen with *n*-propyl coating. The crystallite sizes were estimated to be of the order of around 100 nm (with base diameter and height from 76 nm and 78 nm for bare MCM-41 to 112 nm and 463 nm for MCM-41 lined with *n*-propyl). Although simplified, the considered model of the crystalline microstructure clearly explains the relations between different characteristics and provides new insight into the properties of a class of mesoporous silica-based materials. It has been proven that, in N_2_ physisorption tests, it is extremely important to take into account the chemical composition of the outermost layer of surface atoms of the adsorbent under study.

It would be interesting and useful to calculate the density of porous material from the results of measurements in adsorption analysis. This density is necessary for realistic modelling of the crystalline microstructure and quantitative analysis of the adsorption properties of porous materials such as SBA-15 or MCM-41. At the moment, only the weight of an analysed porous sample is precisely determined, while the volume of its walls is not measured.

## Figures and Tables

**Table 1:** Adsorption characteristics of the four adsorbents tested, estimated from the $\alpha_{s}$-plots with three reference adsorbents and directly from N_2_–physisorption isotherms by BET method; headers: $V_{p}\left[cm^{3}g^{-1}\right]$—the specific volume of mesopores, $S_{t}\left[m^{2}g^{-1}\right]$—total specific surface area, $S_{e}\left[m^{2}g^{-1}\right]$—external specific surface area; $S_{p}=S_{t}-S_{e}\left[m^{2}g^{-1}\right]$—the specific surface area of mesopores, $S_{\mathrm{BET}}\left[m^{2}g^{-1}\right]$—the BET-specific surface area.

Adsorbent Tested	Reference Adsorbent	$V_{p}$; $\left[\begin{array}{c} cm^{3}\\ g^{-1} \end{array}\right]$	$V_{p};\left[\begin{array}{c} cm^{3}STP\\ g^{-1} \end{array}\right]$	$\mathrm{St}S_{t};$ $\left[\begin{array}{c} m^{2}\\ g^{-1} \end{array}\right]$m2g	$S_{e};$ $\left[\begin{array}{c} m^{2}\\ g^{-1} \end{array}\right]$	$S_{p};$ $\left[\begin{array}{c} m^{2}\\ g^{-1} \end{array}\right]$	$S_{\mathrm{BET}};$ $\left[\begin{array}{c} m^{2}\\ g^{-1} \end{array}\right]$
MCM-41 pristine	Nucleosil 1000	0.824	533	892	81.8	810	882
	LiChrospher Si-1000	0.802	518	887	83.0	804	
	Fransil-I	0.807	521	890	85.0	807	
MCM-41-NH_2_	Nucleosil 1000	0.766	495	866	70.6	795	863
	LiChrospher Si-1000	0.756	489	857	68.4	789	
	Fransil-I	0.760	491	862	69.5	792	
MCM-41-SH	Nucleosil 1000	0.707	457	868	72.6	795	861
	LiChrospher Si-1000	0.690	446	856	63.5	793	
	Fransil-I	0.698	451	862	65.1	797	
MCM-41-nC_3_H_7_	Nucleosil 1000	0.655	424	842	53.9	788	834
	LiChrospher Si-1000	0.632	408	840	53.8	786	
	Fransil-I	0.636	411	843	58.7	784	

**Table 2:** The XPS results for the tested samples: binding energies (eV) and elemental compositions (atomic percents) of pristine MCM-41 and MCM-41 lined with organised monolayers of functional molecules, covalently bound to the mesoporous support: -C_3_H_7_, -C_3_H_6_-NH_2_, -C_3_H_6_-SH.

Adsorbent	Element	BE (eV)	%At Conc
MCM-41 (pristine)	C	284.8	13.5
	O	533.1	51.9
	Si	103.3	34.6
MCM-41-NH_2_	C	284.8	10.3
	N	401.1	1.0
	O	533.1	53.0
	Si	103.3	34.7
	Cl	199.3	1.1
MCM-41-SH	C	284.8	16.0
	O	532.3	50.0
	Si	103.3	32.4
	S	167.8	1.6
MCM-41-C_3_H_7_	C	284.8	17.1
	O	533.1	50.8
	Si	103.3	32.1

**Table 3:** Unit cell constant *a* of pristine MCM-41 and modified, calculated from six peaks (symbols in headings are explained in the text).

Adsorbent	a ±∆a	∆a	$d_{100}\pm ∆d_{100}$	$∆d_{100}$	e_r_
MCM-41 (pristine)	4.516 ± 0.077	0.077	3.911 ± 0.066	0.066	0.017
MCM-41-NH_2_	4.517 ± 0.193	0.193	3.912 ± 0.168	0.168	0.043
MCM-41-SH	4.460 ± 0.078	0.078	3.862 ± 0.066	0.066	0.017
MCM-41-nC_3_H_7_	4.499 ± 0.126	0.126	3.896 ± 0.109	0.109	0.028
MCM-41 mean	4.498 ± 0.130	0.130	3.895 ± 0.113	0.113	0.029

**Table 4:** Adsorption characteristics of pristine MCM-41 adsorbents or lined with organized monolayers of functional molecules (symbols in headings are explained in the text).

Adsorbent	S_BET_	${\overset{-}{V}}_{p}$	${\overset{-}{S}}_{t}$	${\overset{-}{S}}_{e}$	${\overset{-}{S}}_{p}$	$\overset{-}{e}$	${\overset{=}{V}}_{p}$	${\overset{=}{S}}_{t}$	${\overset{=}{S}}_{e}$	${\overset{=}{S}}_{p}$	$\overset{=}{e}$
MCM-41 pristine	882	0.811	890	83.3	807	0.0071	0.815	884	84.5	799	0.0100
MCM-41-NH_2_	863	0.761	862	69.5	792	0.0045	0.766	856	70.6	785	0.0100
MCM-41-SH	861	0.698	862	67.1	795	0.0067	0.703	856	68.1	788	0.0100
MCM-41-nC_3_H_7_	834	0.641	842	55.5	786	0.0094	0.645	836	56.4	779	0.0100

**Table 5:** Characteristics of four samples of MCM-41 bare or lined with organized monolayers of functional molecules.

Material	$\bar{a}$	$\bar{w}$	$\hat{w}$	$\overset{=}{w}$	$\overset{=}{\rho }$	$\overset{=}{d}$	${\overset{=}{t}}_{e}$	${\overset{=}{t}}_{i}$	$\overset{=}{L}$	$\overset{=}{H}/\overset{=}{D}$	$\overset{=}{D}$	$\overset{=}{H}$	$\overset{=}{p}$	$\overset{=}{r}$
MCM-41-C16-basic	4.498	2.597	2.147	2.147	2.649	3.719	0.390	0.000	8	1.02	76.5	78.0	0.683	0.911
MCM-41-C16-NH2	4.498	2.597	2.147	2.106	2.490	3.648	0.390	0.036	9	3.35	85.5	286.5	0.656	0.935
MCM-41-C16-SH	4.498	2.597	2.147	2.050	2.318	3.551	0.390	0.084	10	1.95	94.6	184.5	0.620	0.994
MCM-41-C16-nC3H7	4.498	2.597	2.147	1.991	2.179	3.449	0.390	0.135	12	4.11	112.7	463.3	0.584	1.042

## Data Availability

The original contributions presented in the study are included in the article/Supplementary Material, further inquiries can be directed to the corresponding author.

## Supplementary Materials

The following supporting information can be downloaded at: https://www.mdpi.com/article/10.3390/ma17133065/s1, Figure S1: Unsampled pristine MCM-41survey spectra; Figure S2: Survey spectra of the MCM-41-SH sample; Figure S3: Survey spectra of the MCM-41-NH_2_ sample; Figure S4: Survey spectra of the MCM-41-C_3_H_7_ sample; Figure S5: Fitted C1s spectra: pristine MCM-41; MCM-41-SH, MCM-41-NH_2_ and MCM-41-C_3_H_7_; Figure S6: Fitted O1s spectra: MCM-41 pristine, MCM-41-SH, MCM-41-NH_2_ and MCM-41-C_3_H_7_; Figure S7: Fitted Si2p spectra: MCM-41 pristine, MCM-41-SH, MCM-41-NH_2_ and MCM-41-C_3_H_7_; Table S1: Components of fitted C1s spectra. Detailed explanations are given in publications [53,58,59,62]). C-C*–secondary carbon; Table S2: Components of fitted O1s spectra. Si-O: oxygen bonded to carbon, Si-OH: silica hydroxyl groups, and adv. O=C: oxygen double bound to adventitious carbon, adventitious O–C: oxygen single bonded to adventitious carbon [56,60]; Table S3: Components of fitted O1s spectra. Si-O: oxygen bonded to carbon, Si-OH: silica hydroxyl groups, and adv. O=C: oxygen double bound to adventitious carbon, adventitious O–C: oxygen single bonded to adventitious carbon [56,60].

## References

[1] Beck J.S., Vartuli J.C., Roth W.J., Leonowicz M.E., Kresge C.T., Schmitt K.D., Chu C.T.W., Olson D.H., Sheppard E.W., McCullen S.B. (1992). A new family of mesoporous molecular sieves prepared with liquid crystal templates. J. Am. Chem. Soc..

[2] Sing K.S.W., Everett D.H., Haul R.A.W., Moscou L., Pierotti R.A., Rouquerol J., Siemieniewska T. (1985). Reporting physisorption data for gas/solid systems with special reference to the determination of surface area and porosity (Recommendations 1984). Pure Appl. Chem..

[3] Costa J.A.S., de Jesus R.A., Santos D.O., Neris J.B., Figueiredo R.T., Paranhos C.M. (2021). Synthesis, functionalization, and environmental application of silica-based mesoporous materials of the M41S and SBA-n families: A review. J. Environ. Chem. Eng..

[4] Zhao D., Feng J., Huo Q., Melosh N., Fredrickson G.H., Chmelka B.F., Stucky G.D. (1998). Triblock Copolymer Syntheses of Mesoporous Silica with Periodic 50 to 300 Angstrom Pores. Science.

[5] Enache D.F., Vasile E., Simonescu C.M., Culita D., Oprea O., Pandele A.M., Razvan A., Dumitru F., Nechifor G. (2018). Schiff base-functionalized mesoporous silicas (MCM-41, HMS) as Pb(ii) adsorbents. R. Soc. Chem. Adv..

[6] Qin J., Li B., Zhang W., Lv W., Han C., Liu J. (2015). Synthesis, characterization and catalytic performance of well-ordered mesoporous Ni-MCM-41 with high nickel content. Microporous Mesoporous Mater..

[7] MSzeląg M., Janek M., Panek R., Madej J., Fronczyk J. (2022). Modification of the MCM-41 mesoporous silica and its influence on the hydration and properties of a cement matrix. J. Constr. Build. Mat..

[8] Morsi R.E., Mohamed R.S. (2018). Nanostructured mesoporous silica: Influence of the preparation conditions on the physical-surface properties for efficient organic dye uptake. R. Soc. Open Sci..

[9] Prasomsri T., Jiao W., Weng S.Z., Martinez J.G. (2015). Mesostructured zeolites: Bridging the gap between zeolites and MCM-41. R. Soc. Chem. Chem. Commun..

[10] Kresge C.T., Leonowicz M.E., Roth W.J., Vartuli J.C., Beck J.S. (1992). Ordered mesoporous molecular sieves synthesized by a liquid-crystal template mechanism. Nature.

[11] Kumar D., Schumacher K., von Hohenesche C.D.F., Grün M., Unger K. (2001). MCM-41, MCM-48 and related mesoporous adsorbents: Their synthesis and characterisation. Colloids Surf. A Physicochem. Eng. Asp..

[12] Reichinger M. (2007). Poröse Silikate MIT Hierarchischer Porenstruktur: Synthese von Mikro-/Mesoporösem MCM-41 UND MCM-48 Materialien Aus Zeolithischen Baueinheiten Des MFI-Gerüststrukturtyps. Ph.D. Thesis.

[13] Grajek H., Stocki J., Sofińska-Chmiel W., Gąska R., Kojdecki M.A., Puchała M. (2022). Synthesis and investigation of SBA-15 lined with ethylenediamine to create charge-transfer complexes. Microporous Mesoporous Mater..

[14] Yasmin T., Müller K. (2010). Synthesis and surface modification of mesoporous mcm-41 silica materials. J. Chromatogr. A.

[15] Clark J.H., Macquarrie D.J. (1998). Catalysis of liquid phase organic reactions using chemically modified mesoporous inorganic solids. Chem. Commun..

[16] Vallet-Regí M., Colilla M., Izquierdo-Barba I., Manzano M. (2017). Mesoporous Silica Nanoparticles for Drug Delivery: Current Insights. Molecules.

[17] Zhao X.S., Lu G.Q., Whittaker A.K., Millar G.J., Zhu H.Y. (1997). Comprehensive Study of Surface Chemistry of MCM-41 Using 29Si CP/MAS NMR, FTIR, Pyridine-TPD, and TGA. J. Phys. Chem. B.

[18] Lindlar B., Kogelbauer A., Prins R. (2000). Chemical, structural, and catalytic characteristics of Al-MCM-41 prepared by pH-controlled synthesis. Microporous Mesoporous Mater..

[19] Costa J.A.S., de Jesus R.A., Santos D.O., Mano J.F., Romão L.P.C., Paranhos C.M. (2020). Recent progresses in the adsorption of organic, inorganic, and gas compounds by MCM-41-based mesoporous materials. Microporous Mesoporous Mater..

[20] Costa J.A.S., Paranhos C.M. (2020). Mitigation of silica-rich wastes: An alternative to the synthesis eco-friendly silica-based mesoporous materials. Microporous Mesoporous Mater..

[21] Thommes M., Kaneko K., Neimark A.V., Olivier J.P., Rodriguez-Reinoso F., Rouquerol J., Sing K.S.W. (2015). Physisorption of gases, with special reference to the evaluation of surface area and pore size distribution (IUPAC technical report). Pure Appl. Chem..

[22] Chen H., Fu S., Fu L., Yang H., Chen D. (2019). Simple Synthesis and Characterization of Hexagonal and Ordered Al–MCM–41 from Natural Perlite. Minerals.

[23] Yang X., Bai Y., Li Q., Wang J. (2022). Preparation and Adsorption Properties of MCM-41 with Novel Gemini Ionic Liquid Surfactants as Template. Materials.

[24] Martínez-Edo G., Balmori A., Pontón I., del Rio A.M., Sánchez-García D. (2018). Functionalized Ordered Mesoporous Silicas (MCM-41): Synthesis and Applications in Catalysis. Catalysts.

[25] Grün M., Lauer L., Unger K.K. (1997). The synthesis of micrometer- and submicrometer-size spheres of ordered mesoporous oxide MCM-41. Adv. Mater..

[26] Ciesla U., Grün M., Isajeva T., Kurganov A.A., Neimark A.V., Ravikovitch P., Schacht S., Schüth F., Unger K.K., Pinnavaia T.J., Thorpe M.F. (1995). Critical Appraisal of the Pore Structure of MCM-41, in Access in Nanoporous Materials.

[27] Grün M., Kurganov A.A., Schacht S., Schüth F., Unger K.K. (1996). Comparison of an ordered mesoporous aluminosilicate, silica, alumina, titania and zirconia in normal-phase high-performance liquid chromatography. J. Chromatogr. A.

[28] Oberhagemann U., Kinski I., Dierdorf I., Marler B., Gies H. (1996). Synthesis and properties of boron containing MCM-41. J. Non-Cryst. Solids.

[29] Marler B., Oberhagemann U., Vortmann S., Gies H. (1996). Influence of the sorbate type on the XRD peak intensities of loaded MCM-41. Microporous Mater..

[30] Stöber W., Fink A., Bohn E. (1968). Controlled growth of monodisperse silica spheres in the micron size range. J. Colloid Interface Sci..

[31] Monnier A., Schüth F., Huo Q., Kumar D., Margolese D., Maxwell R.S., Stucky G.D., Krishnamurty M., Petroff P., Firouzi A. (1993). Cooperative Formation of Inorganic-Organic Interfaces in the Synthesis of Silicate Mesostructures. Science.

[32] CTripp C.P., Hair M.L. (1995). Direct Observation of the Surface Bonds between Self-Assembled Monolayers of Octadecyltrichlorosilane and Silica Surfaces: A Low-Frequency IR Study at the Solid/Liquid Interface. Langmuir.

[33] Kailasam K., Fels A., Müller K. (2009). Octadecyl grafted MCM-41 silica spheres using trifunctionalsilane precursors—Preparation and characterization. Microporous Mesoporous Mater..

[34] Price P.M., Clark J.H., Macquarrie D.J. (2000). Modified silicas for clean technology. J. Chem. Soc. Dalton Trans..

[35] Chen H., Wang Y. (2002). Preparation of MCM-41 with high thermal stability and complementary textural porosity. Ceram. Int..

[36] Bogush G., Tracy M., Zukoski C. (1988). Preparation of monodisperse silica particles: Control of size and mass fraction. J. Non-Cryst. Solids.

[37] Chen S.-L., Dong P., Yang G.-H. (1997). The Size Dependence of Growth Rate of Monodisperse Silica Particles from Tetraalkoxysilane. J. Colloid Interface Sci..

[38] Chen S.-L., Dong P., Yang G.-H., Yang J.-J. (1996). Characteristic Aspects of Formation of New Particles during the Growth of Monosize Silica Seeds. J. Colloid Interface Sci..

[39] Jin Y., Lohstreter S., Pierce D.T., Parisien J., Wu M., Hall C., Zhao J.X. (2008). Silica Nanoparticles with Continuously Tunable Sizes: Synthesis and Size Effects on Cellular Contrast Imaging. Chem. Mater..

[40] Lee C.-H., Lo L.-W., Mou C.-Y., Yang C.-S. (2008). Synthesis and Characterization of Positive-Charge Functionalized Mesoporous Silica Nanoparticles for Oral Drug Delivery of an Anti-Inflammatory Drug. Adv. Funct. Mater..

[41] Lei Q., Guo J., Noureddine A., Wang A., Wuttke S., Brinker C.J., Zhu W. (2020). Sol–Gel-Based Advanced Porous Silica Materials for Biomedical Applications. Adv. Funct. Mater..

[42] Grajek H., Paciura-Zadrożna J., Choma J., Michalski E., Witkiewicz Z. (2012). Synthesis of OMS Materials and Investigation of Their Acceptor–Donor Characteristics. Chromatographia.

[43] Pearson R.G. (1966). Acids and Bases. Science.

[44] Pearson R.G. (1968). Hard and soft acids and bases, HSAB, part 1: Fundamental principles. J. Chem. Educ..

[45] Pearson R.G. (1968). Hard and soft acids and bases, HSAB, part II: Underlying theories. J. Chem. Educ..

[46] Branton P.J., Hall P.G., Sing K.S.W. (1993). Physisorption of nitrogen and oxygen by MCM-41, a model mesoporous adsorbent. Chem. Soc. Chem. Commun.

[47] Rouquerol F., Rouquerol J., Sing K.S.W. (1999). Adsorption by Powders & Porous Solids.

[48] Jaroniec M., Kruk M., Olivier J.P. (1999). Standard Nitrogen Adsorption Data for Characterization of Nanoporous Silicas. Langmuir.

[49] Qiao S.Z., Bhatia S.K., Zhao X.S. (2003). Prediction of multilayer adsorption and capillary condensation phenomena in cylindrical mesopores. Microporous Mesoporous Mater..

[50] Bhambhani M.R., Cutting P.A., Sing K.S.W., Turk D.H. (1972). Analysis of nitrogen adsorption isotherms on porous and nonporous silicas by the BET and αs methods. J. Colloid Interface Sci..

[51] Sayari A., Liu P., Kruk M., Jaroniec M. (1997). Characterization of Large-Pore MCM-41 Molecular Sieves Obtained via Hydrothermal Restructuring. Chem. Mater..

[52] Villarroel-Rocha J., Barrera D., Blanco A.A.G., Jalil M.E.R., Sapag K. (2013). Importance of the α_s_-plot Method in the Characterization of Nanoporous Materials. Adsorpt. Sci. Technol..

[53] Biesinger M.C. (2022). Accessing the robustness of adventitious carbon for charge referencing (correction) purposes in XPS analysis: Insights from a multi-user facility data review. Appl. Surf. Sci..

[54] Krumpfer J.W., Fadeev A.Y. (2006). Displacement Reactions of Covalently Attached Organosilicon Monolayers on Si. Langmuir.

[55] Fadeev A.Y. (2015). Encyclopedia of Surface and Colloid Science.

[56] Arrigo R., Hävecker M., Wrabetz S., Blume R., Lerch M., McGregor J., Parrott E.P.J., Zeitler J.A., Gladden L.F., Knop-Gericke A. (2010). Tuning the Acid/Base Properties of Nanocarbons by Functionalization via Amination. J. Am. Chem. Soc..

[57] Stobinski L., Lesiak B., Malolepszy A., Mazurkiewicz M., Mierzwa B., Zemek J., Jiricek P., Bieloshapka I. (2014). Graphene oxide and reduced graphene oxide studied by the XRD, TEM and electron spectroscopy methods. J. Electron Spectrosc. Relat. Phenom..

[58] Major G.H., Fairley N., Sherwood P.M.A., Linford M.R., Terry J., Fernandez V., Artyushkova K. (2020). Practical guide for curve fitting in X-ray photoelectron spectroscopy. J. Vac. Sci. Technol. A.

[59] Beamson G., Briggs D. (2000). The XPS of Polymers Database.

[60] McCafferty E., Wightman J.P. (1998). Determination of the concentration of surface hydroxyl groups on metal oxide films by a quantitative XPS method. Surf. Interface Anal..

[61] Rabchinskii M.K., Ryzhkov S.A., Kirilenko D.A., Ulin N.V., Baidakova M.V., Shnitov V.V., Pavlov S.I., Chumakov R.G., Stolyarova D.Y., Besedina N.A. (2020). From graphene oxide towards aminated graphene: Facile synthesis, its structure and electronic properties. Sci. Rep..

[62] Gengenbach T.R., Major G.H., Linford M.R., Easton C.D. (2021). Practical guides for x-ray photoelectron spectroscopy (XPS): Interpreting the carbon 1s spectrum. J. Vac. Sci. Technol. A.

[63] Cerofolini G.F., Galati C., Renna L. (2002). Accounting for anomalous oxidation states of silicon at the Si/SiO_2_ interface. Surf. Interface Anal..

[64] Paparazzo E., Fanfoni M., Severini E. (1992). Studies on the structure of the SiOx/SiO_2_ interface. Appl. Surf. Sci..

[65] Hanke M., Scherzer O. (2001). Inverse Problems Light: Numerical Differentiation. Am. Math. Mon..

[66] Wilson A.J.C. (1980). Relationship between ‘observed’ and ‘true’ intensity; effect of various counting modes. Acta Crystallogr..

[67] Luger P. (1980). X-ray Analysis of Single Crystals.

[68] Kojdecki M.A. (2001). Deconvolution by Example—Computational Test of Effective Algorithms. Mater. Sci. Forum.

[69] Corriu R.J.P., Lancelle-Beltran E., Mehdi A., Reyé C., Brandès S., Guilard R. (2002). Ordered mesoporous hybrid materials containing cobalt(ii) Schiff base complex. J. Mater. Chem..

[70] Morozov V.A. (1984). Methods for Solving Incorrectly Posed Problems.

[71] Tikhonov A.N., Goncharsky A.V., Stepanov V.V., Yagola A.G. (1995). Numerical Methods for the Solution of Ill-Posed Problems.

[72] Miloslavsky V.K., Makovetsky E.D., Ageev L.A., Beloshenko K.S. (2009). Fused silica as a composite nanostructured material. Opt. Spectrosc..

[73] Prezhdo O., Drogosz A., Zubkova V., Prezhdo V. (2013). Density of normal and associated liquids. Fluid Phase Equilibria.

[74] Fryxell G.E., Mattigod S.V., Lin Y., Wu H., Fiskum S., Parker K., Zheng F., Yantasee W., Zemanian T.S., Addleman R.S. (2007). Design and synthesis of self-assembled monolayers on mesoporous supports (SAMMS): The importance of ligand posture in functional nanomaterials. J. Mater. Chem..

[75] Smith E.A., Chen W. (2008). How To Prevent the Loss of Surface Functionality Derived from Aminosilanes. Langmuir.

[76] Lee L.H. (1991). Fundamentals of Adhesion.

