# Supplementary material for: Parametric Modelling of the Crystalline Microstructure of the MCM41-Type Mesoporous Silica Modified with Derivatives of Alkyls
Source: Materials (Basel). 2024 Jun 21;17(13):3065. doi: 10.3390/ma17133065 (PMC11242262; doi:10.3390/ma17133065)
Supplement: Supplementary file 1 [file materials-17-03065-s001.zip › materials-3043992-supplementary.pdf]

materials

Supporting Information

Parametric Modelling of the Crystalline Microstructure of the MCM41-Type Mesoporous Silica Modified with Derivatives of Alkyls

Jarosław Stocki [1,*], Marcin Kuśmierz [2], Weronika Sofińska-Chmiel [2], Marek Stankevič [3], Marcin Puchała [1], Marek A. Kojdecki [4], Robert Gąska [1] and Henryk Grajek [1]

[1] Faculty of Advanced Technologies and Chemistry, Military University of Technology, 00-908 Warsaw, Poland; hgrajek5819@wp.pl (H.G.)
[2] Analytical Laboratory, Institute of Chemical Sciences, Faculty of Chemistry, Maria Curie Skłodowska University in Lublin, 20-031 Lublin, Poland; marcin.kusmierz@mail.umcs.pl (M.K.)
[3] Department of Organic Chemistry and Crystallochemistry, Institute of Chemical Sciences, Faculty of Chemistry, Maria Curie-Skłodowska University in Lublin, 20-614 Lublin, Poland
[4] Institute of Mathematics and Cryptology, Military University of Technology, 00-908 Warsaw, Poland
* Correspondence: jaroslaw.stocki@wat.edu.pl

XPS results

Individual XPS survey spectra of the examined materials: MCM-41, MCM-41-SH, MCM-41-NH_2 and MCM-41-C_3H_7:

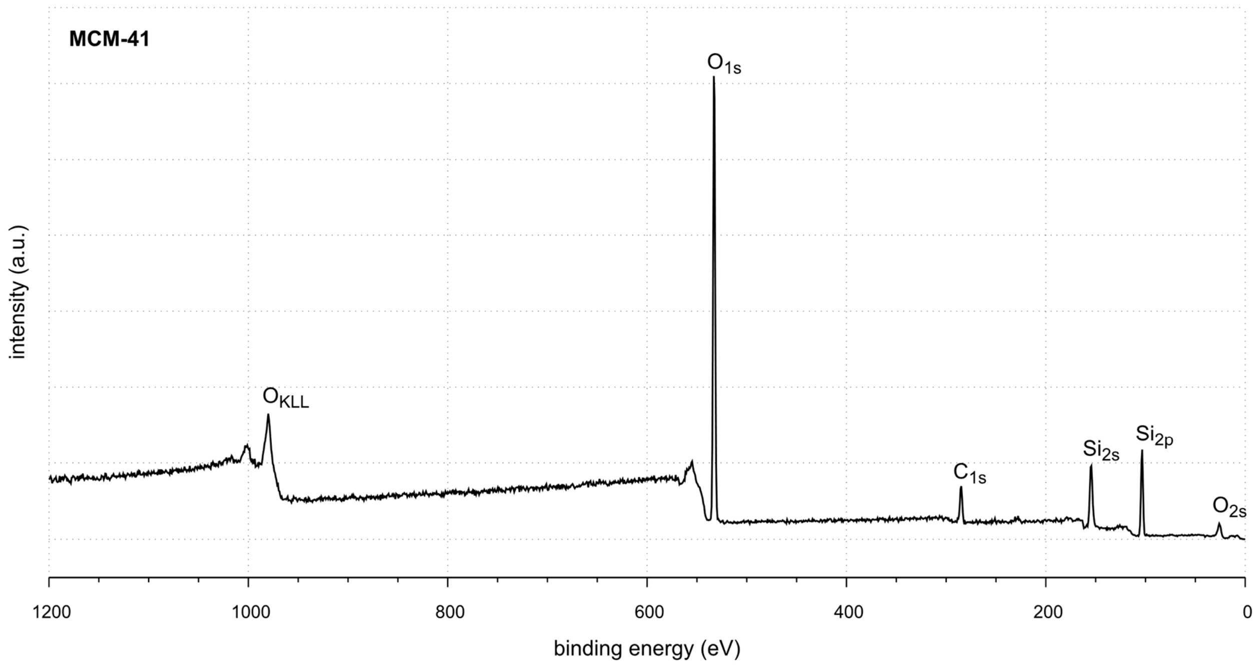

Figure S1. Unsampled pristine MCM-41survey spectra.

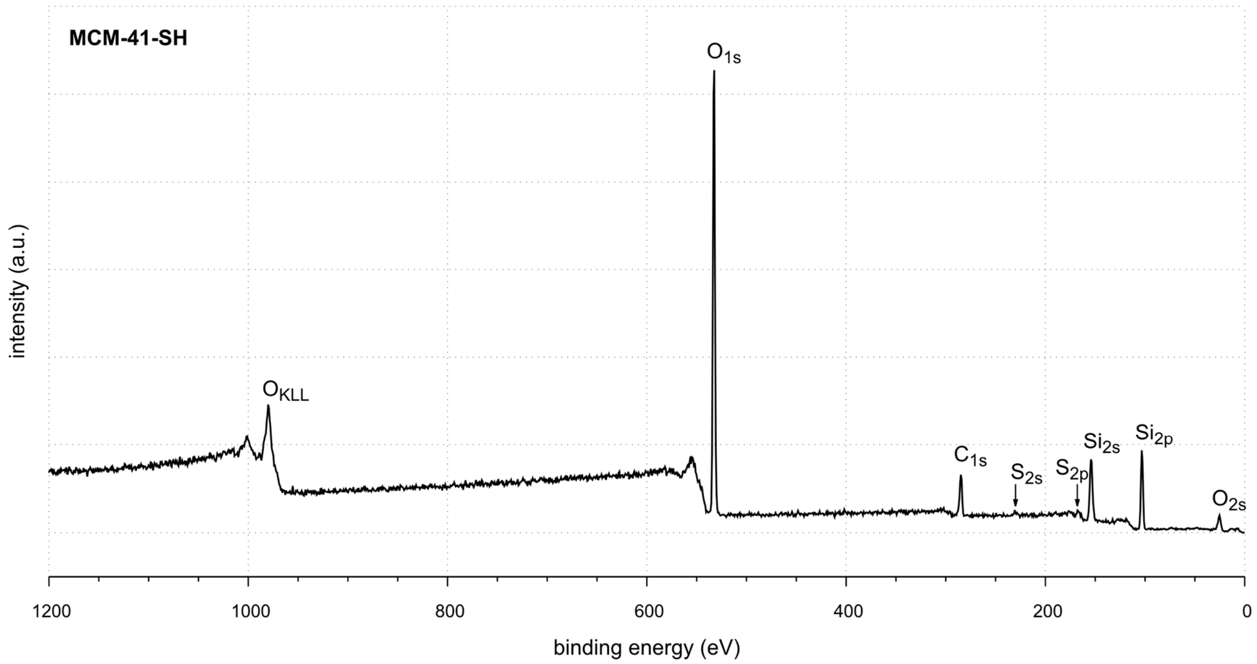

Figure S2. Survey spectra of the MCM-41-SH sample.

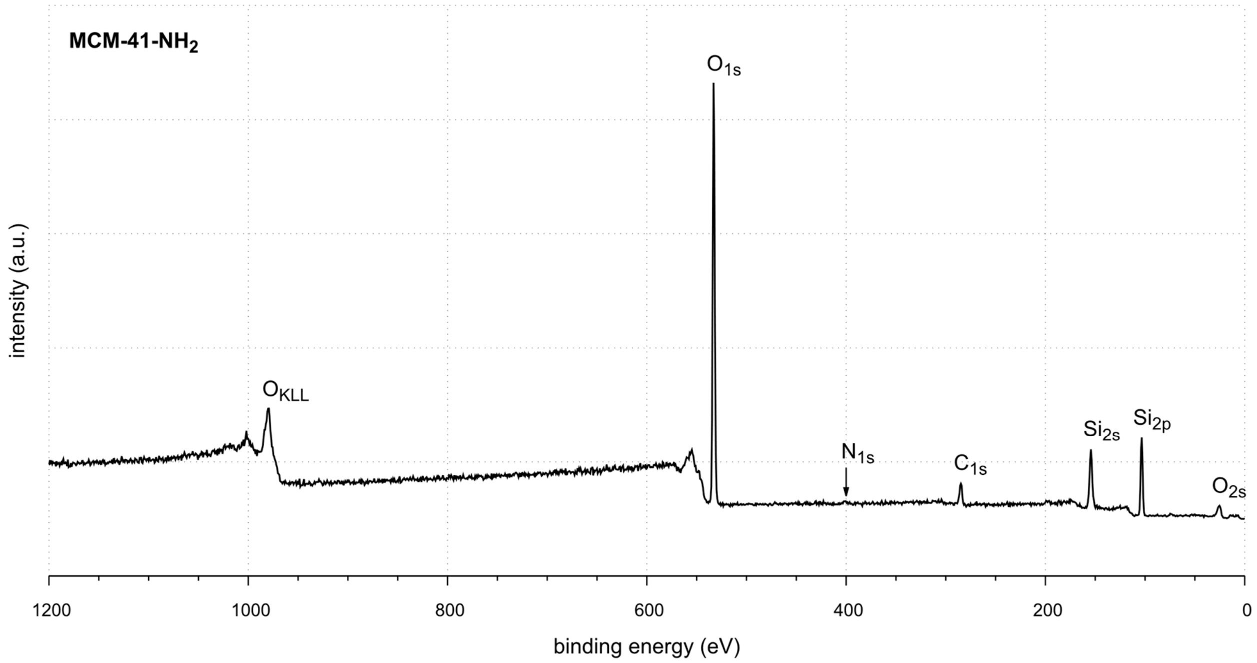

Figure S3. Survey spectra of the MCM-41-NH_2 sample.

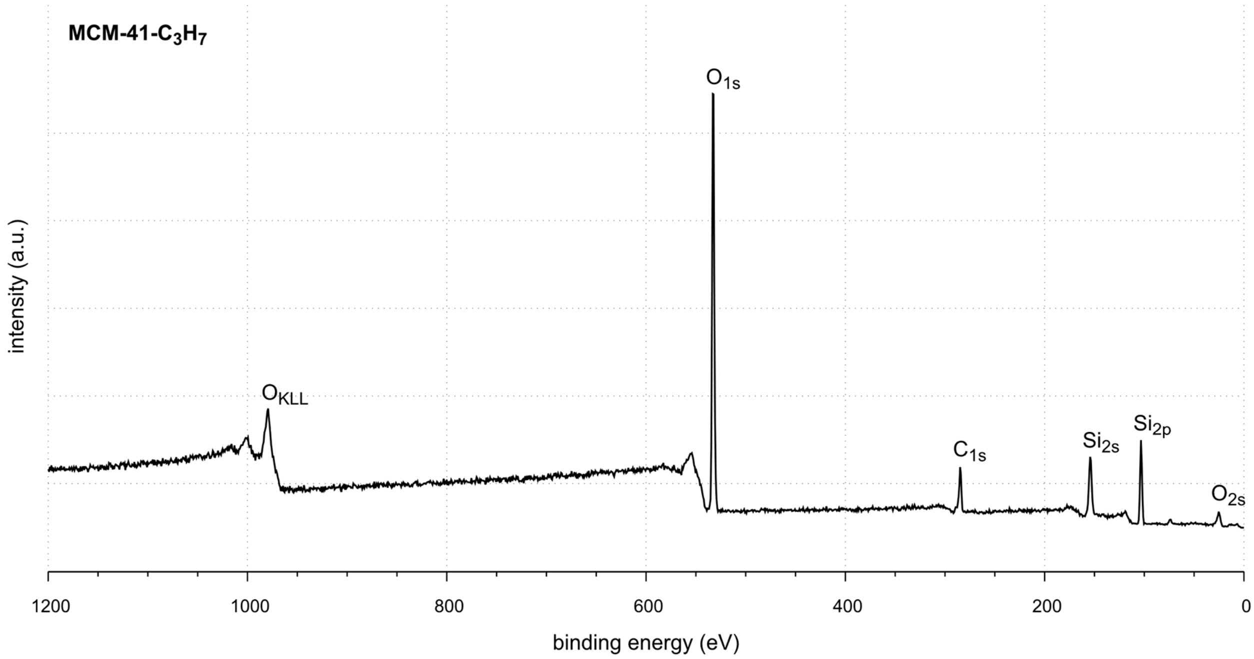

Figure S4. Survey spectra of the MCM-41-C_3H_7 sample.

Fitted C1s spectra of the examined materials: pristine MCM-41, MCM-41-SH, MCM-41-NH_2 and MCM-41-C_3H_7. The C1s region model was based on publications [55,60,61,63,64], C-C* - secondary carbon.

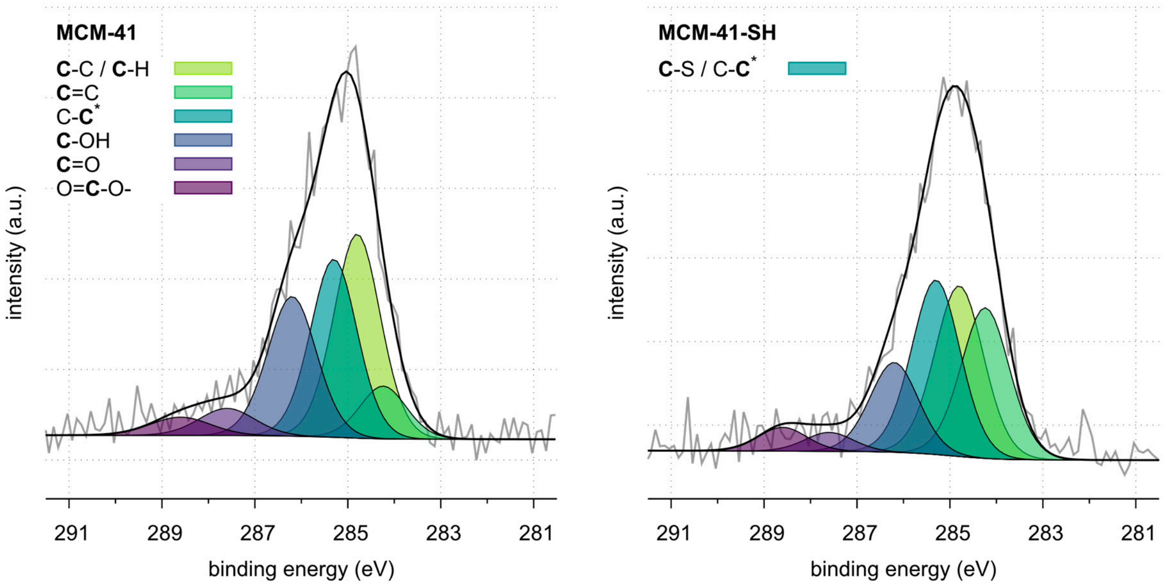

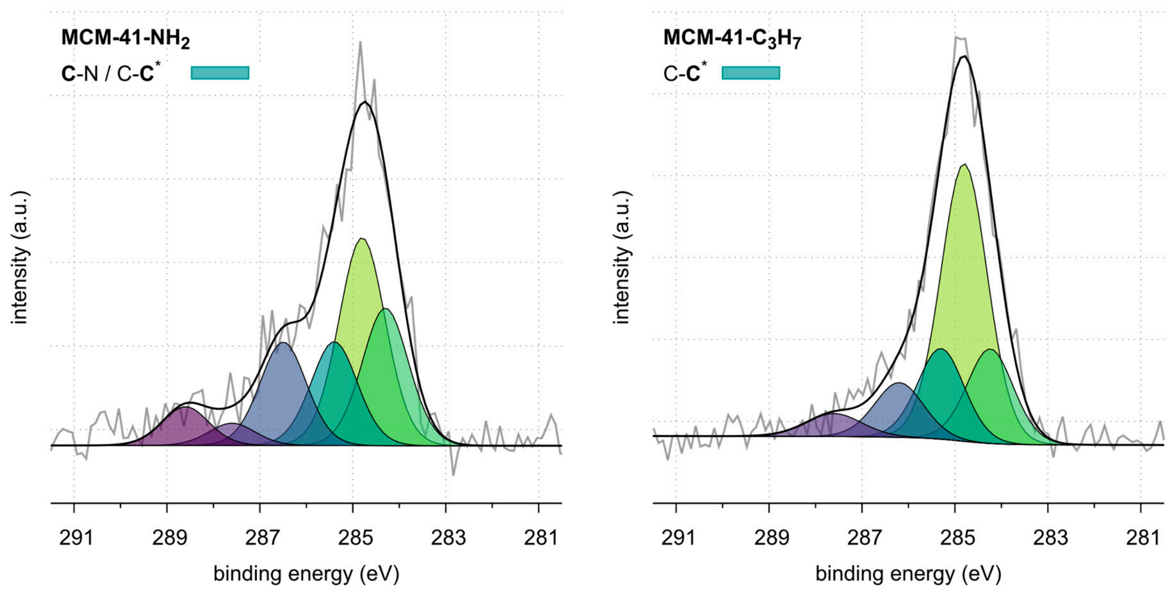

Figure S5. Fitted C1s spectra: pristine MCM-41, MCM-41-SH, MCM-41-NH_2 and MCM-41-C_3H_7.

Table S1. Components of fitted C1s spectra. Detailed explanations are given in publications [55,60,61,64]). C-C* –secondary carbon.

Sample	Component	BE (eV)	%At. Conc.
MCM-41 (pristine)	C-C / C-H	284.80	32.0
	C=C sp^2	284.24	8.3
	C-C*	285.30	27.9
	C-OH	286.20	23.0
	C=O	287.60	5.1
	COOH	288.60	3.7
MCM-41-C_3H_7	C-C / C-H	284.80	50.7
	C=C sp^2	284.24	17.3
	C-C*	285.30	16.6
	C-OH	286.20	10.5
	C=O	287.60	5.0
	COOH	288.60	0.0
MCM-41-NH_2	C-C / C-H	284.80	33.9
	C=C sp^2	284.30	22.4
	C-N / C-C*	285.40	16.9
	C-OH	286.50	16.9

	C=O	287.60	3.6
	COOH	288.60	6.3
MCM-41-SH	C-C / C-H	284.80	27.2
	C=C sp^2	284.24	24.0
	C-S / C-C*	285.30	27.8
	C-OH	286.20	14.3
	C=O	287.60	3.0
	COOH	288.60	3.8

Fitted O1s spectra of the examined materials: MCM-41 pristine, MCM-41-SH, MCM-41-NH_2 and MCM-41-C_3H_7. The O1s region model was based on publications [58,59,62].

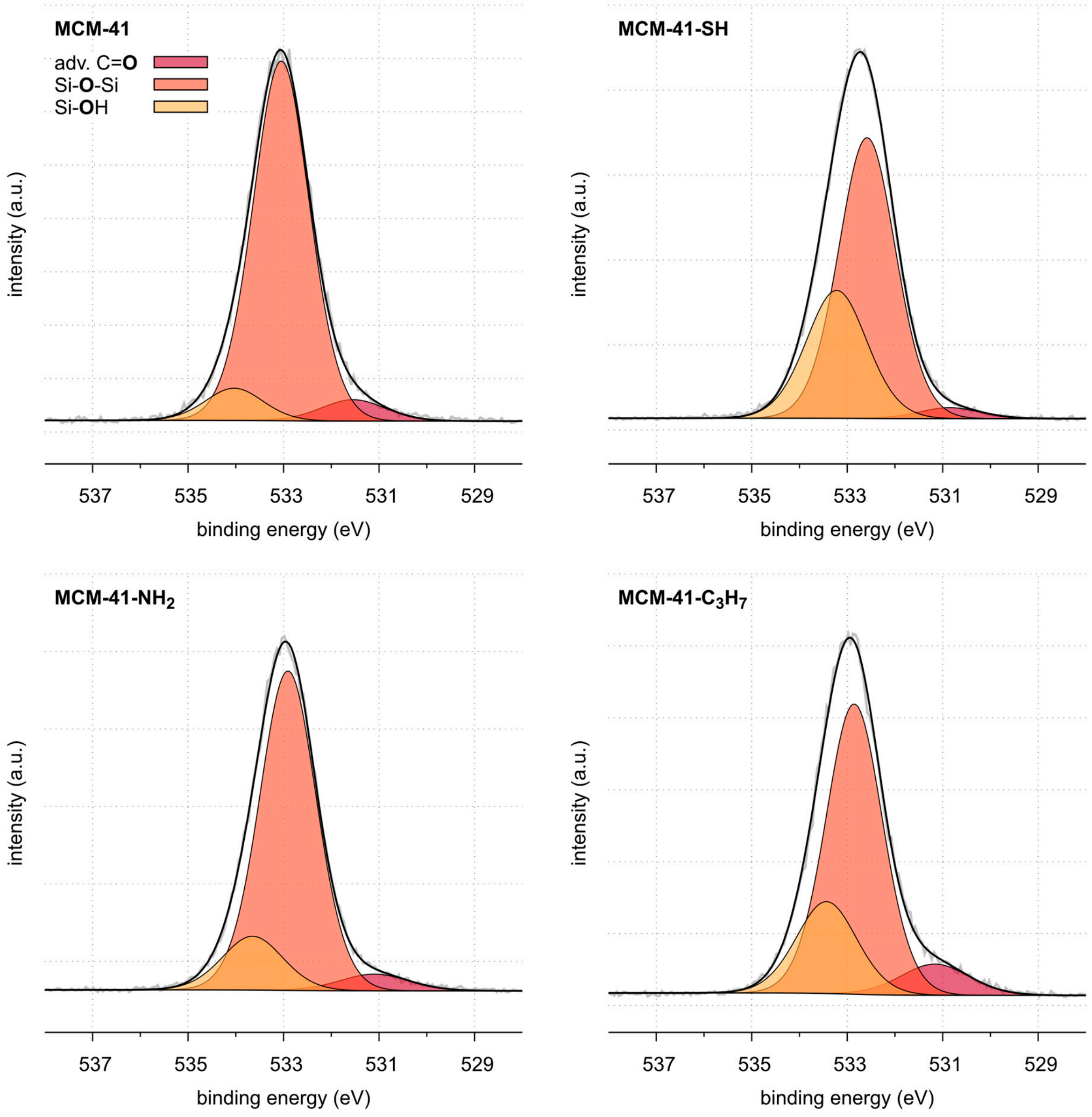

Figure S6. Fitted O1s spectra: MCM-41 pristine, MCM-41-SH, MCM-41-NH_2 and MCM-41-C_3H_7.

Table S2. Components of fitted O1s spectra. Si-O: oxygen bonded to carbon, Si-OH: silica hydroxyl groups, and adv. O=C: oxygen double bound to adventitious carbon, adventitious O - C: oxygen single bonded to adventitious carbon [58,62].

Sample	Component	BE (eV)	%At. Conc.
MCM-41 (pristine)	Si-O	532.98	86.2
	adventitious O=C	531.48	5.9
	Si-OH / adventitious O-C	533.97	7.9
MCM-41-C_3H_7	Si-O	532.98	68
	adventitious O=C	531.29	8.9
	Si-OH / adventitious O-C	533.56	23.1
MCM-41-NH_2	Si-O	532.94	80.4
	adventitious O=C	531.14	5.1
	Si-OH / adventitious O-C	533.69	14.5
MCM-41-SH	Si-O	532.87	65.4
	adventitious O=C	531.14	2.9
	Si-OH / adventitious O-C	533.50	31.7

Fitted Si2p spectra of the examined materials: MCM-41 pristine, MCM-41-SH, MCM-41-NH_2 and MCM-41-C_3H_7. The Si2p region model was based on publications [65,66].

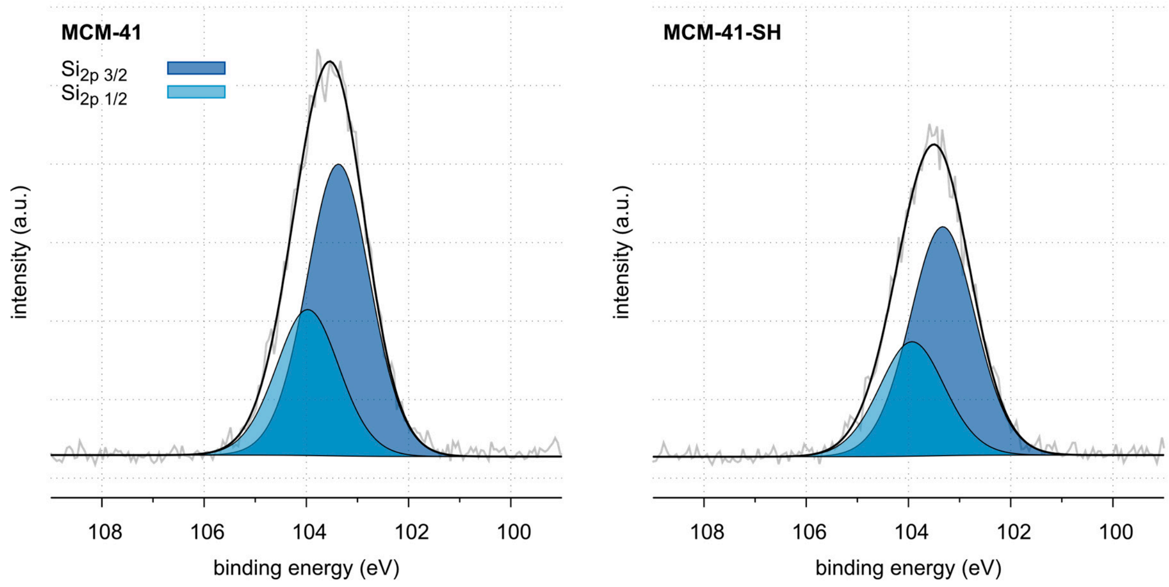

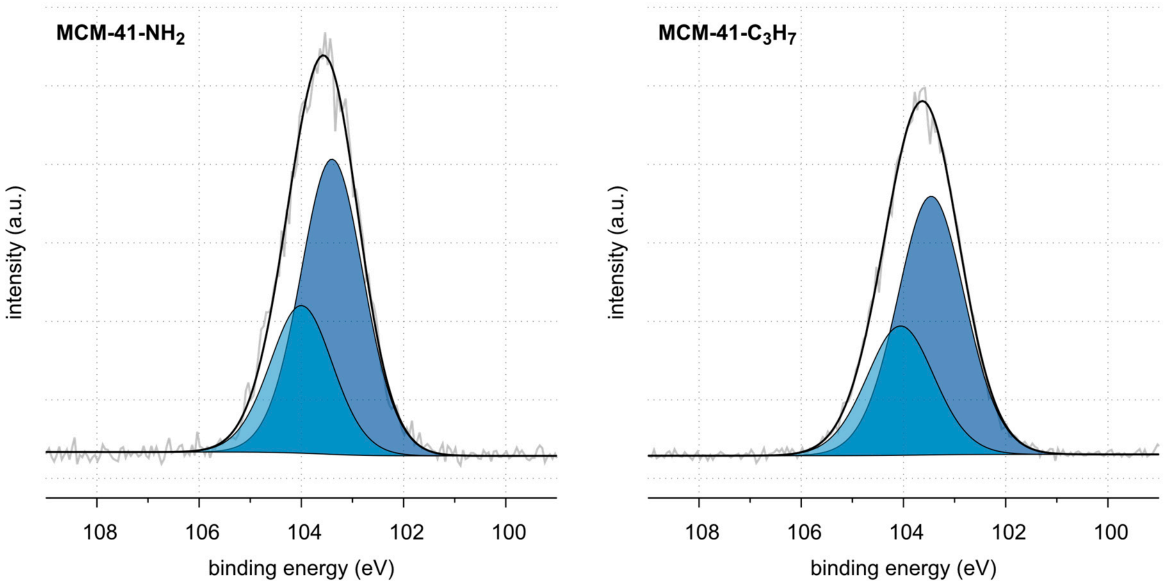

Figure S7. Fitted Si2p spectra: MCM-41 pristine, MCM-41-SH, MCM-41-NH_2 and MCM-41-C_3H_7.

Table S3. Components of fitted O1s spectra. Si-O: oxygen bonded to carbon, Si-OH: silica hydroxyl groups, and adv. O=C: oxygen double bound to adventitious carbon, adventitious O - C: oxygen single bonded to adventitious carbon [58,62].

Sample	Component	BE (eV)	%At. Conc.
MCM-41 (pristine)	Si 2p 3/2	103.37	100.0
	Si 2p 1/2	103.97	
MCM-41-C3H7	Si 2p 3/2	103.46	100.0
	Si 2p 1/2	104.06	
MCM-41-NH2	Si 2p 3/2	103.40	100.0
	Si 2p 1/2	104.00	
MCM-41-SH	Si 2p 3/2	103.33	100.0
	Si 2p 1/2	103.93	
